# Identification of topoisomerase 2A as a novel bone metastasis-related gene in liver hepatocellular carcinoma

**DOI:** 10.18632/aging.205216

**Published:** 2023-11-17

**Authors:** Jinyan Feng, Xianfu Wei, Yongheng Liu, Yanting Zhang, Guanghao Li, Yao Xu, Peng Zhou, Jin Zhang, Xiuxin Han, Chao Zhang, Yan Zhang, Guowen Wang

**Affiliations:** 1 Department of Bone and Soft Tissue Tumors, Tianjin Medical University Cancer Institute and Hospital, National Clinical Research Center for Cancer, Tianjin, China; 2 Key Laboratory of Cancer Prevention and Therapy, Tianjin, China; 3 Tianjin’s Clinical Research Center for Cancer, Tianjin, China; 4 Department of Orthopedics, Affiliated Hospital of Chifeng University, Chifeng, China

**Keywords:** liver hepatocellular carcinoma, bone metastasis, topoisomerase 2A, hippo-YAP pathway, prognosis

## Abstract

Background: Bone is the second most frequent site of metastasis for Liver hepatocellular carcinoma (LIHC), which leads to an extremely poor prognosis. Identifying novel biomarkers and therapeutic targets for LIHC patients with bone metastasis is urgently needed.

Methods: In this study, we used multiple databases for comprehensive bioinformatics analysis, including TCGA, GEO, ICGC, GTEx, TISIDB, and TIMER, to identify key genes related to bone metastasis of LIHC. Clinical tissues and tissue microarray were adopted to assess the expression of TOP2A through qRT-PCR and immunohistochemistry analyses in LIHC. Gene enrichment analysis, DNA methylation, gene mutation, prognosis, and tumor immunity associated with TOP2A in LIHC were investigated. *In vitro* and *in vivo* experiments were performed to explore the functional role of TOP2A in LIHC bone metastasis.

Results: We identified that TOP2A was involved in LIHC bone metastasis. Clinically, TOP2A was highly expressed in LIHC tumoral specimens, with the highest level in the bone metastasis lesions. TOP2A was an independent prognostic factor that higher expression of TOP2A was markedly associated with poorer prognosis in LIHC. Moreover, the abnormal expression of TOP2A might be related to DNA hypomethylation, often accompanied by TP53 mutation, immune escape and immunotherapy failure. Enrichment analysis and validation experiments unveiled that TOP2A stimulated the Hippo-YAP signaling pathway in LIHC. Functional assays confirmed that TOP2A could promote bone-specific metastatic potential and tumor-induced osteolysis in LIHC.

Conclusions: These findings unveil that TOP2A might be a novel prognostic biomarker and therapeutic target for LIHC bone metastasis.

## INTRODUCTION

Liver hepatocellular carcinoma (LIHC) is the main histological subtype of liver cancer, accounting for 90% of primary liver cancers [1, 2]. Although significant progress has been made in the treatment of LIHC in recent years, the overall prognosis of LIHC patients remains devastating. Only ~ 20% of LIHC patients can be treated with radical surgery, and the probability of postoperative recurrence and metastasis is 60-70%, resulting in a 5-year overall survival rate of less than 10% for LIHC [3, 4]. Remarkably, 25.5% ~ 38.5% of LIHC patients will develop bone metastasis, second only to lung metastasis, which seriously affects the quality of life and prognosis of LIHC patients, whose median survival is only about 7.4 months [5–7]. Up to now, the molecular mechanism of bone metastasis of LIHC has not been fully elucidative, which poses great challenges to its treatment. Therefore, it is imperative to deeply explore the pathogenesis of bone metastasis of LIHC and identify potential novel biomarkers and therapeutic targets for early diagnosis and prognosis of LIHC patients with bone metastasis.

Topoisomerase 2A (TOP2A) is a key member of the type II DNA topoisomerase family, which is mainly involved in DNA division, repair, recombination, replication, transcription, and chromosome separation and condensation by regulating DNA topology [8, 9]. The TOP2A coding gene, located at 17q12-21, functions as the target of several anticancer agents, and various mutations in this gene are related to the development of drug resistance [10]. A growing number of studies have shown that TOP2A is correlated to the occurrence, development, metastasis, and prognosis of various tumors, including lung cancer, breast cancer, cervical cancer, bladder cancer, and so on [11–14]. Studies have also found that TOP2A is abnormally expressed in LIHC and may be a factor affecting its prognosis [15]. Nevertheless, the role and molecular mechanism of TOP2A in bone metastasis of LIHC have not been reported and need to be further explored.

In this study, by mining various databases for comprehensive analysis, we identified that TOP2A might be a bone metastasis-related gene for LIHC. TOP2A was highly enriched in LIHC tumoral tissues, especially in the bone metastasis lesions and might serve as a potential prognostic biomarker. Moreover, TOP2A as the key regulator in DNA replication and repair might promote tumor aggression by affecting the genetic integrity of TP53, further resulting in the dysregulation of cell processes. Functionally, TOP2A could promote the Hippo-YAP signaling pathway, and was involved in tumor cell growth, bone-specific metastatic potential and tumor-induced osteolysis in LIHC. In conclusion, our study sheds light on the significance of TOP2A in LIHC bone metastasis and provides a new target and biomarker for the treatment and prognosis of LIHC bone metastasis.

## RESULTS

### TOP2A is associated with bone metastasis of LIHC

**Figure 1:**
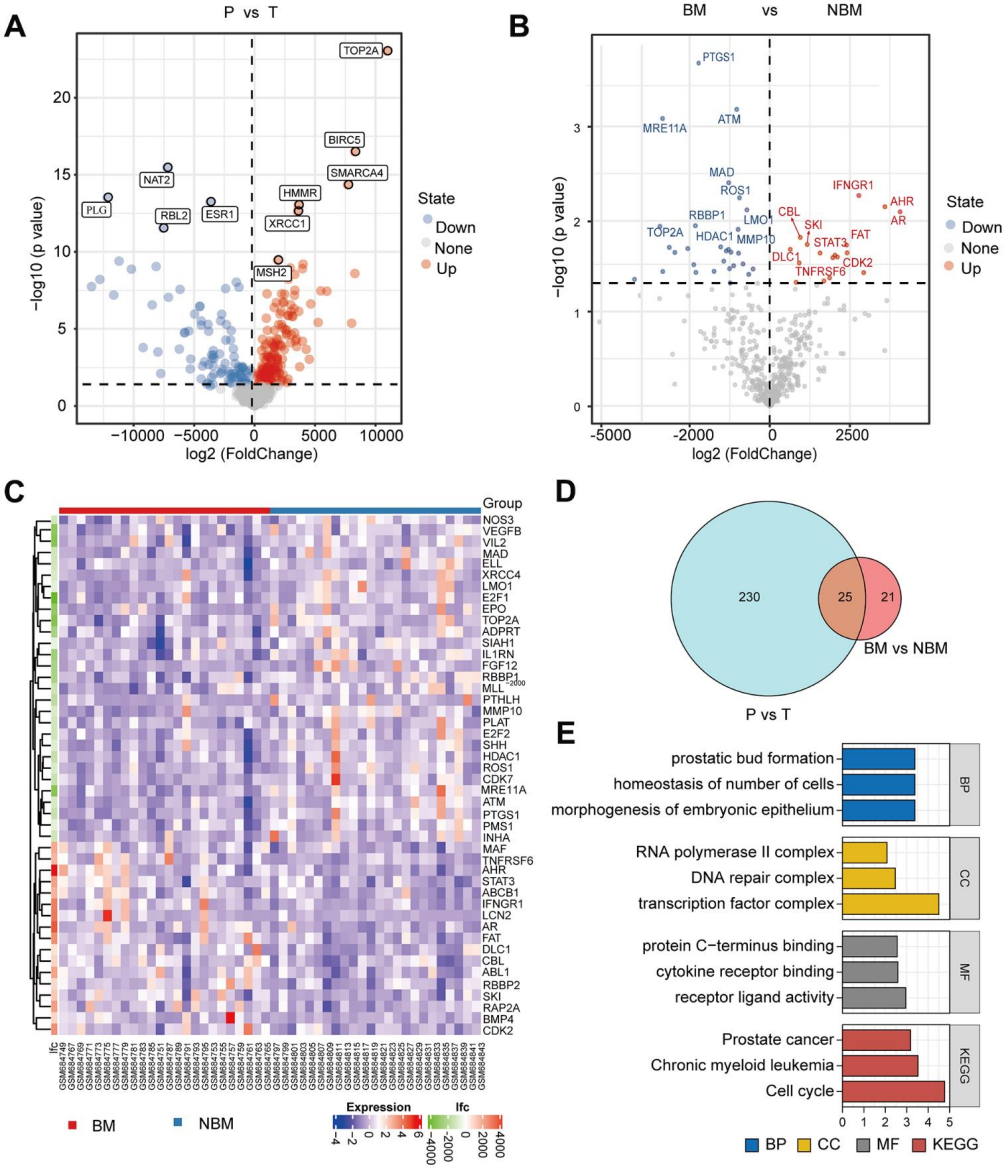
**TOP2A is associated with bone metastasis of liver hepatocellular carcinoma.** (**A**) Volcano plot of differentially expressed genes (DEGs) between LIHC and paracancerous tissues from GSE27635 database, red indicated up-regulated genes, and blue indicated down-regulated genes. (**B**) Volcano plot of DEGs between LIHC with BM and NBM. (**C**) Heatmap of DEGs between LIHC with and without BM. (**D**) Venn diagram of DEGs from A and B. (**E**) GO and KEGG enrichment analysis of the above overlapping genes. P, peritumor; T, tumor; BM, bone metastasis; NBM, non-bone metastasis.

The mRNA expression data and clinical data of 48 matched tumoral and peritumoral tissues from LIHC patients, including 24 patients with bone metastasis (BM), were retrieved from the GSE27635 database. Firstly, volcano plot showed the DEGs between 48 tumoral and peritumoral tissues (Figure 1A). Meanwhile, the DEGs between LIHC patients with or without BM and their counterpart tissues were displayed, respectively (Supplementary Figure 1A, 1B). Interestingly, TOP2A, BIRC5, and SMARCA were all significantly up-regulated, while NAT2, PLG, and ESR1 were remarkably down-regulated. Among them, the upregulation of TOP2A was most significant in the 3 groups. Moreover, Figure 1B, 1C revealed that a total of 46 genes had differential expressions between LIHC patients with and without BM. Next, we used Venn diagrams (Figure 1D) to identify overlapping BM-related genes with the above DEGs from LIHC and paracancerous tissues, and DEGs from BM and non-bone metastasis (NBM) LIHC. There were 25 overlapping BM-related genes, including TOP2A. To further explore the function of the 25 overlapping BM-related genes, GO enrichment analyses and KEGG pathway analyses were conducted. As shown in Figure 1E, the overlapping BM-related genes were enriched in prostate bud formation, morphogenesis of embryonic epithelium, and homeostasis of number of cells in terms of biological process. In the category of cellular component, the above genes were associated with RNA polymerase II transcription factor complex, DNA repair complex, and transcription factor complex. Additionally, in the analysis of the molecular function, protein C-terminus binding, cytokine receptor binding, and receptor ligand activity were the most enriched terms mediated by the overlapping genes. KEGG pathways analysis revealed that the above BM-related genes were mainly related to prostate cancer, chronic myeloid leukemia, and cell cycle. Pathway analysis further illustrated the relationship between 25 overlapping BM-related genes and cancer-related pathways, showing that TOP2A was closely associated with the activity of EMT and cell cycle pathway (Supplementary Figure 1C). As TOP2A displayed great significance in expression and correlation with LIHC bone metastasis, we selected it for the next research.

### Aberrant expression of TOP2A in LIHC patients

**Figure 2:**
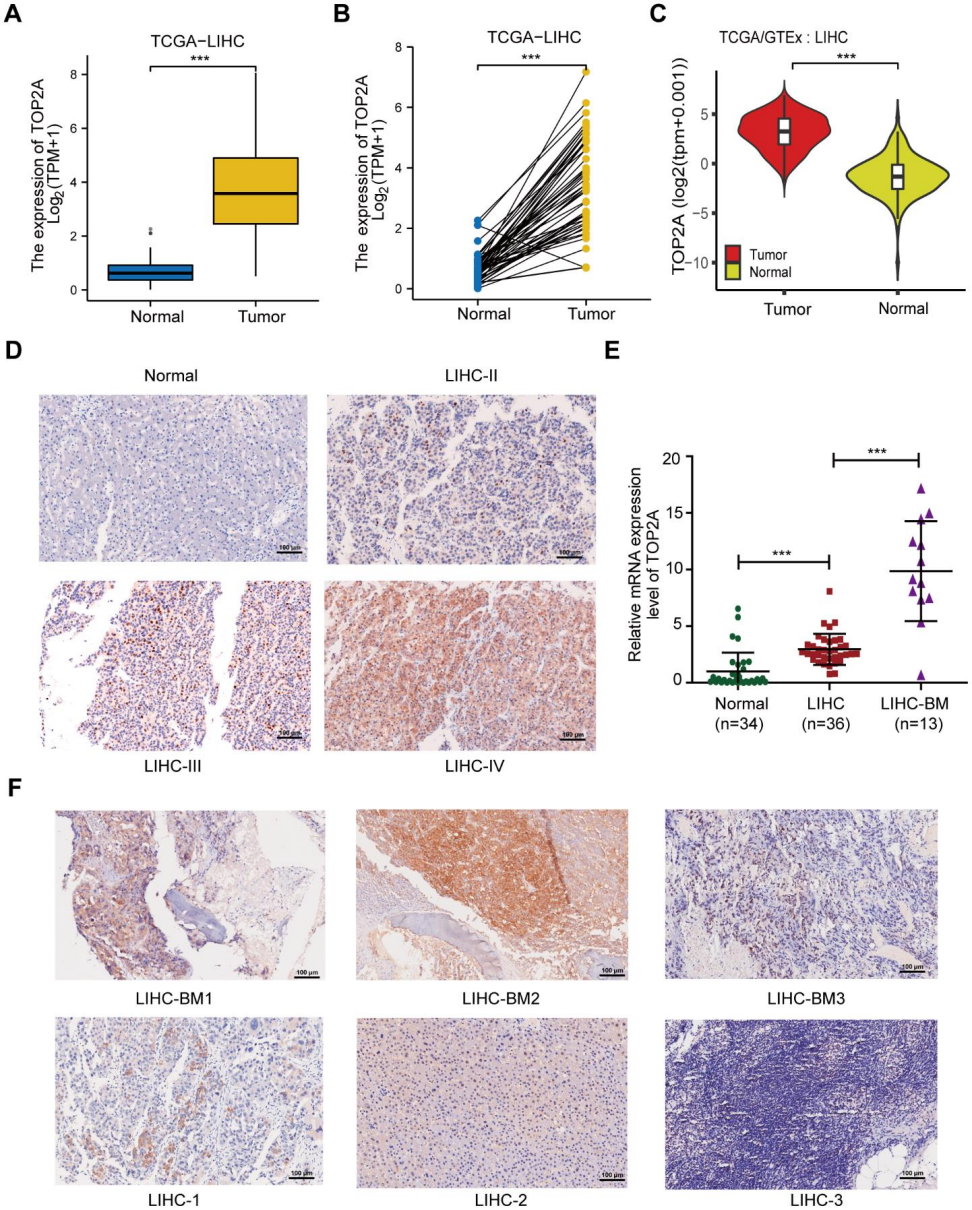
**The expression of TOP2A in LIHC.** (**A**) The mRNA expression level of TOP2A of LIHC and normal tissues in TCGA database. (**B**) TOP2A mRNA expression of matched LIHC and normal tissues in TCGA database. (**C**) The mRNA expression level of TOP2A of LIHC and normal tissues in TCGA and GTEx database. (**D**) IHC of TOP2A protein expression in LIHC with different stage and normal liver tissues from tissue microarrays. (**E**) The mRNA expression level of TOP2A of LIHC, LIHC-BM and normal liver tissues in Tianjin Medical University Cancer Institute and Hospital. (**F**) IHC of TOP2A protein expression in three paired LIHC-BM lesions and counterpart primary LIHC tissues from Tianjin Medical University Cancer Institute and Hospital. Magnification: 200 ×, scale bars: 100 μm. ***P < 0.001.

In order to further explore the role of TOP2A in LIHC, we first identified its mRNA and protein levels in LIHC. The results showed that the transcriptional level of TOP2A was significantly elevated in LIHC tissues compared with normal liver tissues or their matched adjacent normal tissues (Figure 2A–2C). Moreover, consistent results appeared in 10 GEO datasets, confirming the high expression of TOP2A in LIHC (Table 1). Then, we further analyzed the protein expression level of TOP2A through the HPA database. Supplementary Figure 2A revealed that TOP2A protein expression level was upregulated in LIHC with high or medium staining, but not detected in normal liver tissues. Moreover, we purchased the LIHC tissue microarray, consisting of 16 normal liver samples and 79 cases of LIHC that included 42 cases in TNM stage II, 30 cases in stage III, and 2 cases in stage IV, while others were unknown. Immunohistochemistry (IHC) analysis showed that the protein expression level of TOP2A increased with the progression of clinical staging, with the highest expression in stage IV, higher expression in stage III, and lower expression in stage II, all of which were higher than those in the normal liver tissues (Figure 2D and Supplementary Figure 2B). Besides, correlation analysis between TOP2A expression and clinical characteristics of LIHC patients from TCGA revealed a remarkable association between high expression of TOP2A and clinical staging, histologic grade, and T stage in LIHC (Supplementary Figure 2C–2E). Taken together, these findings suggest that TOP2A is overexpressed at both mRNA and protein levels in LIHC patients.

**Table 1:** TOP2A expression in HCC and adjacent tissues from 10 GEO datasets.

**Dataset**	***P*-value**	**Type**	**Nums**	**Mean**	**STD**	**IQR**
GSE22058	**8.12E-58**	HCC	100	9.621	1.206	1.69
		Adjacent	97	5.998	0.9358	1.228
GSE25097	**1.79E-54**	HCC	268	2.072	1.588	1.943
		Adjacent	243	0.1474	0.1324	0.104
		Cirrhotic	40	0.2757	0.2667	0.214
		Healthy	6	0.064	0.0343	0.03075
GSE36376	**3.88E-92**	HCC	240	8.611	1.229	1.878
		Adjacent	193	6.074	0.3752	0.4299
GSE14520	**5.03E-86**	HCC	225	6.709	1.401	1.949
		Adjacent	220	3.826	0.4401	0.3011
GSE10143	**4.59E-19**	HCC	80	12.29	0.9772	0.9992
		Adjacent	82	10.8	0.8789	1.361
GSE46444	**0.03166**	HCC	88	7.83	2.564	4.342
		Adjacent	48	6.972	1.97	3.859
GSE54236	**1.13E-12**	HCC	81	6.759	1.882	2.176
		Adjacent	80	4.804	1.216	1.205
GSE63898	**1.90E-52**	HCC	228	6.05	1.211	1.892
		Adjacent	168	4.438	0.2391	0.3067
GSE64041	**9.78E-15**	HCC	60	7.2	1.145	1.8
		Adjacent	60	5.665	0.5444	0.4367
GSE76427	**6.08E-16**	HCC	115	9.804	1.669	1.64
		Adjacent	52	7.926	0.9899	1.385

Next, to further explore the relationship between TOP2A expression and bone metastasis in LIHC, we collected clinical samples from our center, including 34 normal liver tissues, 36 LIHC tissues, and 13 bone metastasis tissues. qRT-PCR assay illustrated that TOP2A displayed the highest expression in LIHC bone metastasis lesions, followed by LIHC tumor tissues, while the expression level was the lowest in normal liver specimens (Figure 2E), indicating that TOP2A was involved in the LIHC bone metastasis. Additionally, we collected three paired LIHC-BM lesions and counterpart primary LIHC tumors from our center for IHC analysis (Figure 2F). The results showed that compared with the corresponding primary tumors, the protein expression level of TOP2A was elevated in LIHC bone metastatic lesions. The above findings indicate that TOP2A expression is closely related to the carcinogenesis and progression of LIHC, including bone metastasis.

### Prognostic value of TOP2A in LIHC patients

**Figure 3:**
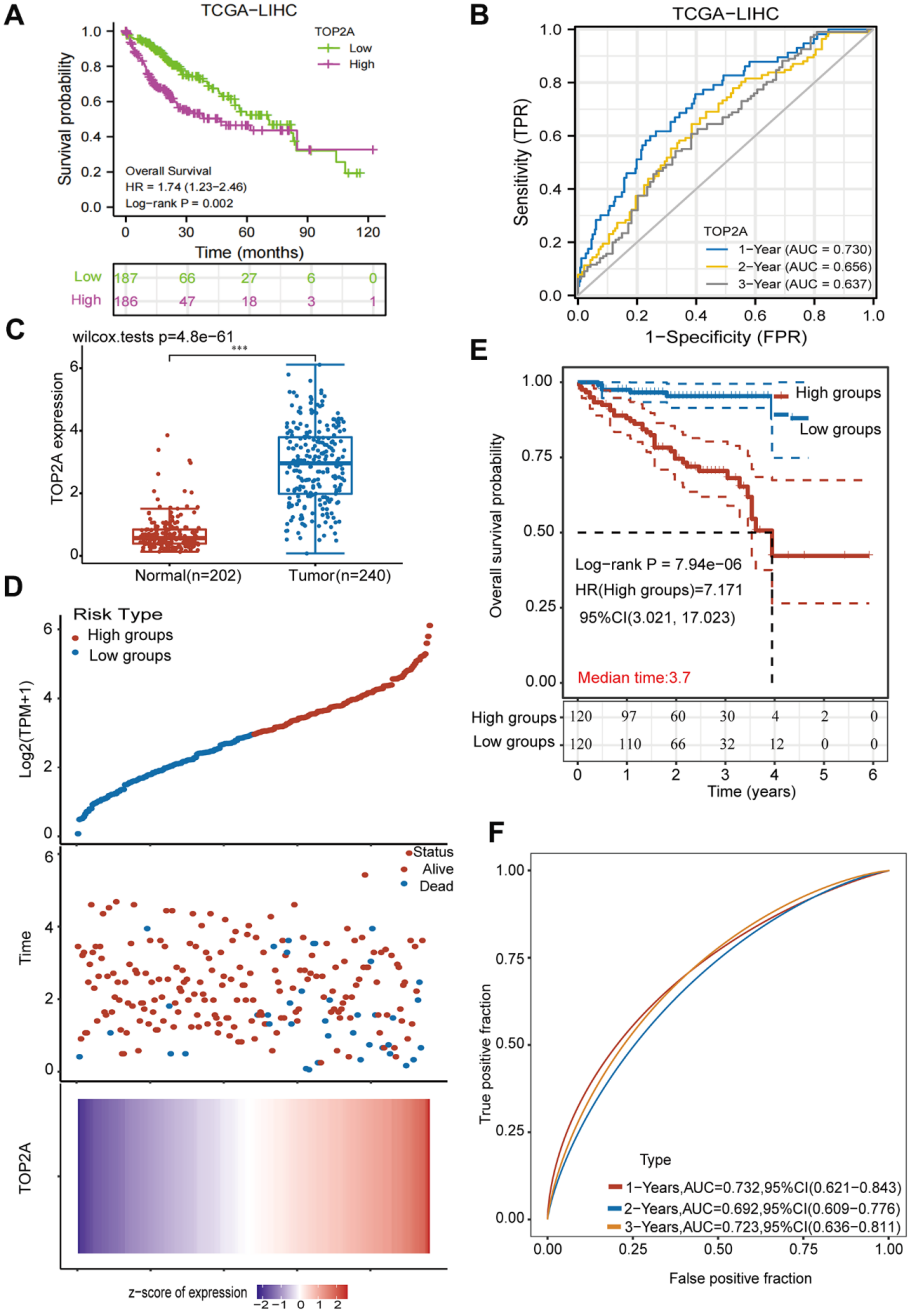
**The prognostic value of TOP2A in LIHC.** (**A**) The overall survival curve of TOP2A in LIHC. (**B**) ROC curves of TOP2A to predict the sensitivity and specificity of 1-, 2-, and 3-year overall survival. (**C**) The mRNA expression level of TOP2A of LIHC and normal tissues in ICGC database. (**D**) Distribution of risk score, survival status, and TOP2A expression profile of LIHC in ICGC database. (**E**) Kaplan-Meier curve of overall survival in high-and low-risk groups. (**F**) ROC curve of the TOP2A risk score model. HR, hazard ratio. ***P < 0.001.

After exploring the characteristics of TOP2A expression levels, we evaluated the prognostic value of TOP2A mRNA expression in LIHC from the TCGA database using Kaplan–Meier plotter. Our results suggested that higher mRNA levels of TOP2A were associated with poorer OS, DSS, and PFI in LIHC patients (Figure 3A and Supplementary Figure 3A, 3B). In addition, the time-dependent ROC curves of TOP2A were applied to predict OS, DSS, and PFI. The AUC for 1-year survival was the largest, while it decreased with the years, for 3-year survival being the smallest, but all values exceeded 0.55, which identified a better survival prediction ability of TOP2A (Figure 3B and Supplementary Figure 3C, 3D). To further explore whether TOP2A was an independent prognostic factor in LIHC patients, a multivariable Cox proportional hazard model was conducted using the TIMER database. The results confirmed that TOP2A was an independent risk factor for clinical outcomes in LIHC patients (HR 1.233, 95%CI 1.044-1.458, *P* =0.014) (Table 2). We further validated the expression characteristics and prognostic value of TOP2A in LIHC from the ICGC database. Consistently, TOP2A was overexpressed in LIHC (Figure 3C). LIHC patients in the ICGC cohort were divided into high-risk (n = 120) and low-risk groups (n = 120) based on the medium cutoff point. The distribution of risk score, survival status, and expression of TOP2A of each LIHC patient was shown in Figure 3D. Kaplan-Meier curve showed that patients in the high-risk group had worse OS (Figure 3E, HR 7.171, 95% CI 3.021-17.02, *P* < 0.0001). Time-dependent ROC curves revealed a better predictive ability of TOP2A in LIHC (Figure 3F). Taken together, TOP2A is highly expressed in LIHC and indicates poor prognosis, which may serve as a potential prognostic biomarker.

**Table 2:** A multivariable Cox proportional hazard model for LIHC using TIMER web server.

**Parameters**	**coef**	**HR**	**(95% CI)**	***P* value**
Age	0.017	1.017	0.999-1.035	0.061
Gender male	0.005	1.005	0.618-1.634	0.983
stage2	0.127	1.136	0.663-1.944	0.643
stage3	0.721	2.056	1.261-3.354	0.004**
stage4	1.674	5.336	1.506-18.899	0.009**
raceBlack	1.206	3.340	1.214-9.183	0.019 *
raceWhite	-0.031	0.970	0.581-1.619	0.907
Purity	0.412	1.510	0.464-4.919	0.494
B_cell	-7.274	0.001	0.000-1.015	0.050
CD8_T cell	-4.682	0.009	0.000-1.406	0.068
CD4_T cell	-8.139	0.000	0.000-0.484	0.031*
Macrophage	8.633	5613.908	21.202-1486464.45	0.002**
Neutrophil	-5.016	0.007	0.000-798.859	0.401
Dendritic	4.270	71.543	1.492-3430.303	0.031*
TOP2A	0.210	1.233	1.044-1.458	0.014 *

Model: Surv(LIHC) ~ Age + Gender + Stage + Race + Purity + B_cell + CD8_Tcell + CD4_Tcell + Macrophage + Neutrophil + Dendritic + TOP2A 305 patients with 101 dying.

HR, hazards ratio; CI, confidence interval. *P<0.05. Significant p-values given in bold.

### TOP2A expression levels and prognostic analysis in pan-cancer

To explore the role of TOP2A in cancers, we further evaluated TOP2A expression in various tumors and adjacent normal tissues. TCGA and GTEx data illustrated that TOP2A expression was significantly upregulated in most cancer types, such as BLCA, CESC, BRCA, CHOL, COAD, ESCA, GBM, HNSC, KICH, KIRC, KIRP, LAML, LIHC, LGG, LUAD, LUSC, OV, PCCG, PRAD, PEAD, SKCM, STAD, TGCT, THCA, UCEC, and UCS, suggesting that TOP2A might function as an oncogene in diverse tumors (Supplementary Figure 4A, 4B). Additionally, the differential expression levels of TOP2A were observed between various tumors and metastatic tumors compared with their matched normal tissues. As the tumor progressed, the expression level of TOP2A was also increased in LUAD, LIHC, SKCM, PRAD, BRCA, and KIRC tumors (Supplementary Figure 4C–4H). Next, we further investigated the prognostic value of TOP2A in pan-cancer using the TCGA database. The results showed that elevated expression of TOP2A was significantly associated with poorer OS in KIRP, LGG, KIRC, MESO, KICH, ACC, LIHC, PAAD, LUAD, and UVM (HR > 1, *P*-value <0.05). While TOP2A had no prognostic values in other types of cancers (HR ≤ 1, *P*-value > 0.05) (Supplementary Figure 5). Collectively, these findings indicate that TOP2A involved in tumor carcinogenesis and progression might be a prognosis biomarker in pan-cancer, especially in LIHC.

### DNA methylation alterations of TOP2A and gene mutation associated with TOP2A in LIHC

**Figure 4:**
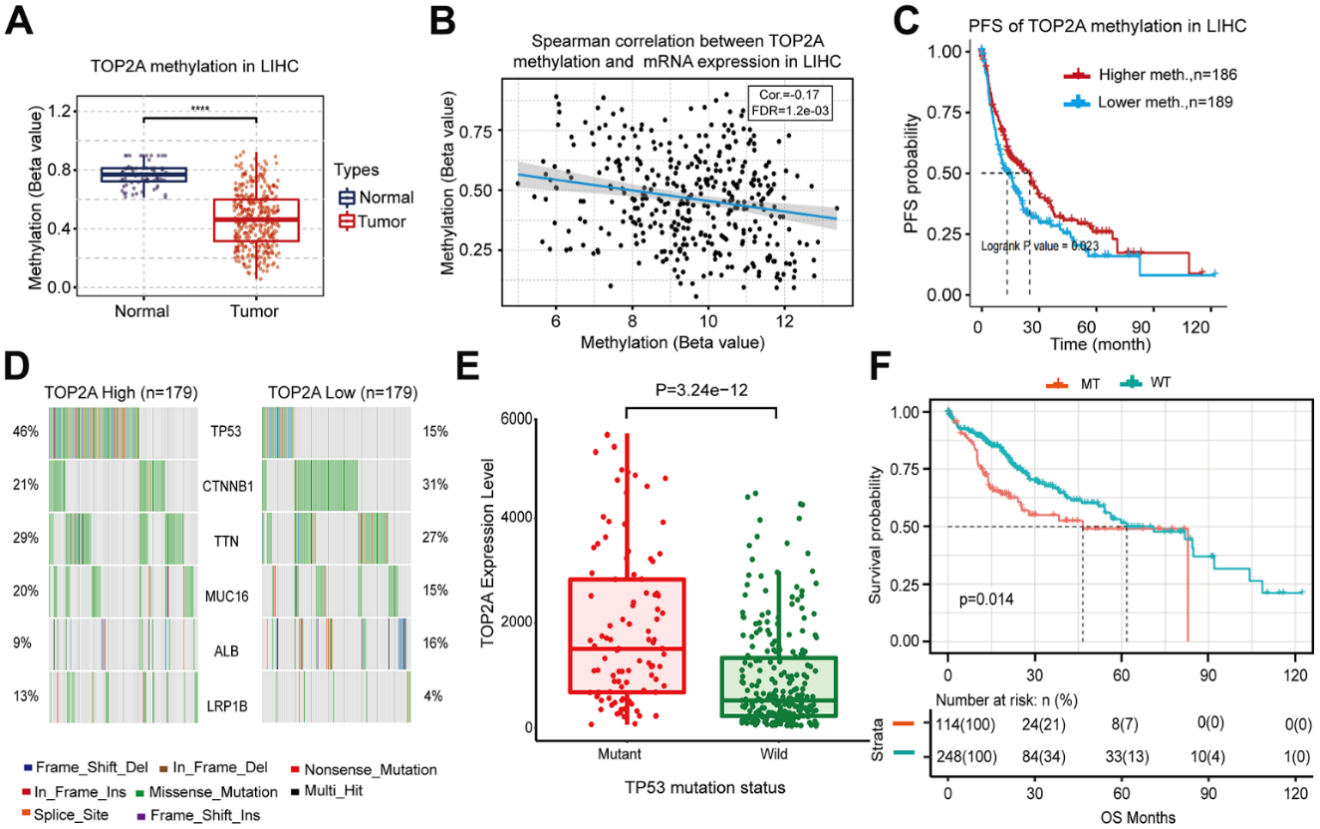
**DNA methylation alterations of TOP2A and gene mutation analysis associated TOP2A in LIHC from TCGA database.** (**A**) The methylation level of TOP2A in LIHC. (**B**) Spearman correlation between DNA methylation and mRNA expression of TOP2A in LIHC. (**C**) Progress-free survival (PFS) curve of TOP2A methylation in LIHC. (**D**) Gene mutations associated with TOP2A in LIHC. (**E**) TOP2A expression level in different TP53 mutant status in LIHC. (**F**) Overall survival (OS) curve of TP53 mutation in LIHC. ***P < 0.001.

It is well known that genetic and epigenetic changes play a key role in the occurrence and development of cancer. First of all, DNA methylation analysis of TOP2A in LIHC was explored using the TCGA database. As shown in Figure 4A, the DNA methylation level of the TOP2A gene promoter in tumor tissues was significantly lower than that in their adjacent normal tissues. Spearman correlation analysis further revealed a negative correlation between DNA methylation and TOP2A mRNA expression levels in LIHC (Figure 4B), implying that DNA hypomethylation might be an important reason for the high expression of TOP2A in LIHC. Moreover, survival analysis showed that lower DNA methylation of TOP2A was associated with poorer survival (Figure 4C). These results suggest that TOP2A is regulated by epigenetic DNA methylation, which is associated with tumor development in LIHC.

Next, we found that LIHC patients with high TOP2A expression were mainly accompanied by 6 genes mutation, including TP53, CTNNB1, TTN, MUC16, ALB, and LRP1B. Among them, the mutation frequency of TP53 was the highest in LIHC with higher expression of TOP2A, in which missense mutation accounted for the main mutation type (Figure 4D). Therefore, we further explored the relationship between p53 mutation and TOP2A expression. The results showed that TOP2A mRNA expression levels were higher in TP53-mutated LIHC than those in TP53-wild LIHC (Figure 4E), implying that TOP2A as the key regulator in DNA replication and repair might promote tumor aggression by affecting the genetic integrity of TP53. Further survival analysis suggested that TP53 mutation was associated with a poor prognosis of LIHC (Figure 4F). In summary, TOP2A is up-regulated by gene promoter hypomethylation and associated with TP53 gene mutation, which leads to poorer prognosis in LIHC.

### Potential function and mechanism analysis of TOP2A in LIHC

**Figure 5:**
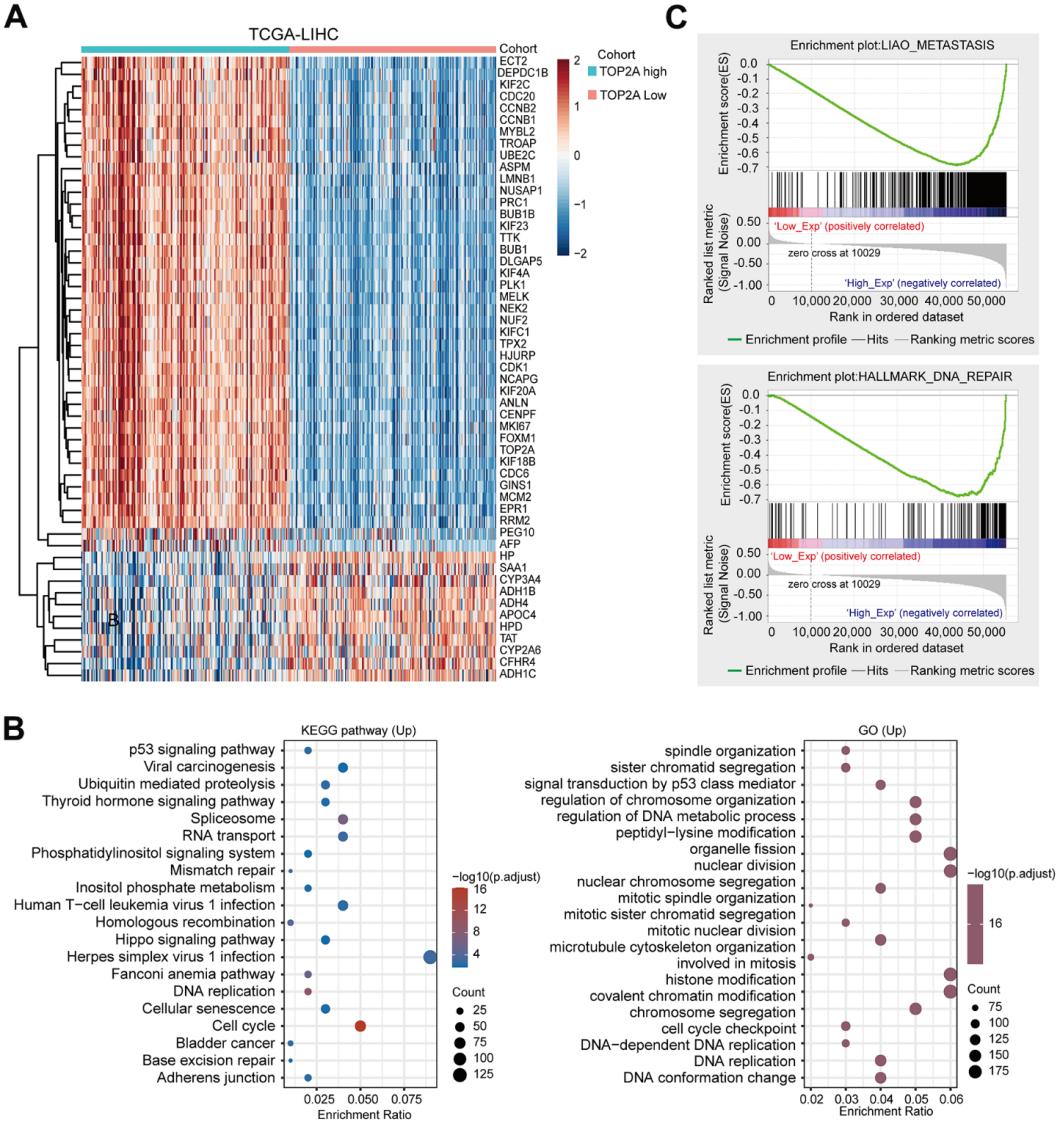
**Functional enrichment analysis of TOP2A in LIHC.** (**A**) Heatmap of TOP2A co-expressed genes in LIHC using TCGA database. (**B**) KEGG and GO enrichment analysis for the up-regulated genes in LIHC. (**C**) Gene Set Enrichment Analysis (GSEA) for TOP2A in LIHC.

Since TOP2A played an important role in LIHC, we further explored its underlying biological function and molecular mechanism. First, co-expressed genes with TOP2A were identified in LIHC using the TCGA database, and the heatmap showed the top 53 genes positively or negatively related to TOP2A in LIHC (Figure 5A). Then the up-regulated or down-regulated genes were separately performed GO and KEGG analyses, to figure out the molecular mechanisms by which TOP2A regulated oncogenesis. KEGG pathway analysis revealed that the up-regulated genes were significantly associated with herpes simplex virus 1 infection, cell cycle, viral carcinogenesis, spliceosome, human T−cell leukemia virus 1 infection, Hippo-YAP signaling pathway, and adherens junction, etc. (Figure 5B, Left). Intriguingly, the Hippo-YAP pathway has been reported to regulate the balance between osteoblastic bone formation and osteoclast bone resorption, and promote bone metastasis [16, 17]. Additionally, the adherens junction was linked to EMT and metastasis [18]. The above results suggest that TOP2A may be involved in bone metastasis of liver cancer. Furthermore, GO analysis showed that these upregulated genes were primarily associated with signal transduction by p53 class mediator, regulation of DNA metabolic process, nuclear division, histone modification, covalent chromatin modification, cell cycle checkpoint, DNA replication, and so on, which all were related to tumor proliferation and epigenetic regulation (Figure 5B, Right). Meanwhile, KEGG analysis revealed that down-regulated genes were mainly enriched in complement and coagulation cascades, retinol metabolism, metabolism of xenobiotics by cytochrome P450, chemical carcinogenesis, and other metabolic-related pathways (Supplementary Figure 6A). GO enrichment analysis displayed that down-regulated genes were primarily connected with the small molecule catabolic process, carboxylic acid biosynthetic process, fatty acid metabolic process, organic acid biosynthetic process, carboxylic acid biosynthetic process, and alcohol metabolic process (Supplementary Figure 6B). GSEA further identified that gene sets related to metastasis, cell cycle, and DNA repair were enriched in LIHC with high expression of TOP2A, suggesting that TOP2A was positively correlated with metastasis and cell cycle in LIHC (Figure 5C and Supplementary Figure 6C).

**Figure 6:**
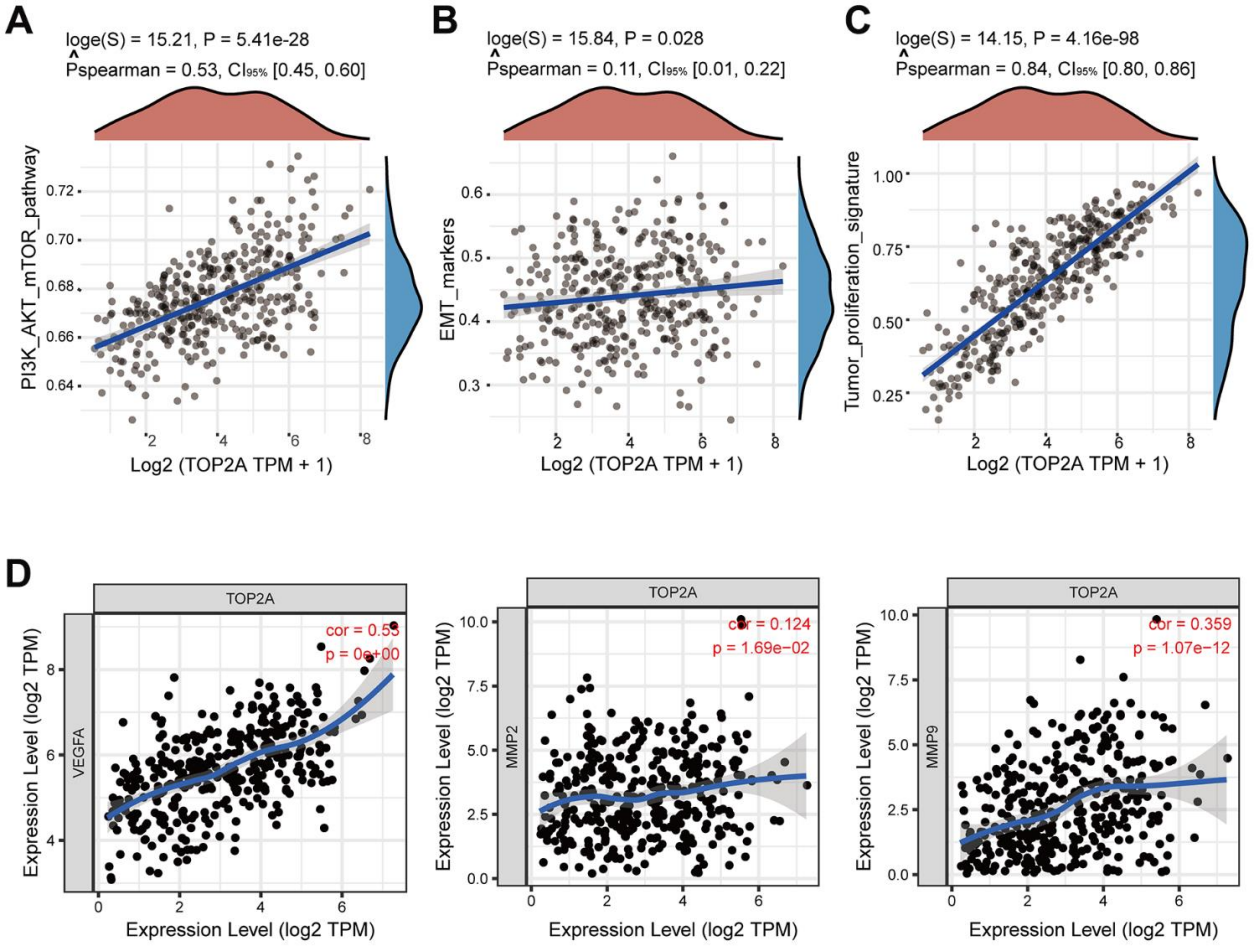
**Correlation analysis between TOP2A expression and pathways and markers related to metastasis or proliferation using TCGA database.** (**A**–**C**) Spearman correlation analysis between TOP2A and PI3K-AKT-mTOR pathway, EMT markers, and tumor proliferation signature. (**D**) Spearman correlation analysis between the expression level of TOP2A and VEGFA, MMP2, and MMP9.

More importantly, correlation analysis showed a positive relationship between TOP2A expression and PI3K-AKT-mTOR pathway, EMT markers, and tumor proliferation signature (Figure 6A–6C). Along with that, TOP2A was positively correlated with metastasis-related genes like VEGFA, MMP2, and MMP9 (Figure 6D). Taken together, these results suggest that TOP2A may be involved in the occurrence and progression of LIHC through multiple pathways.

### Immune infiltration and immunotherapy related to TOP2A expression

**Figure 7:**
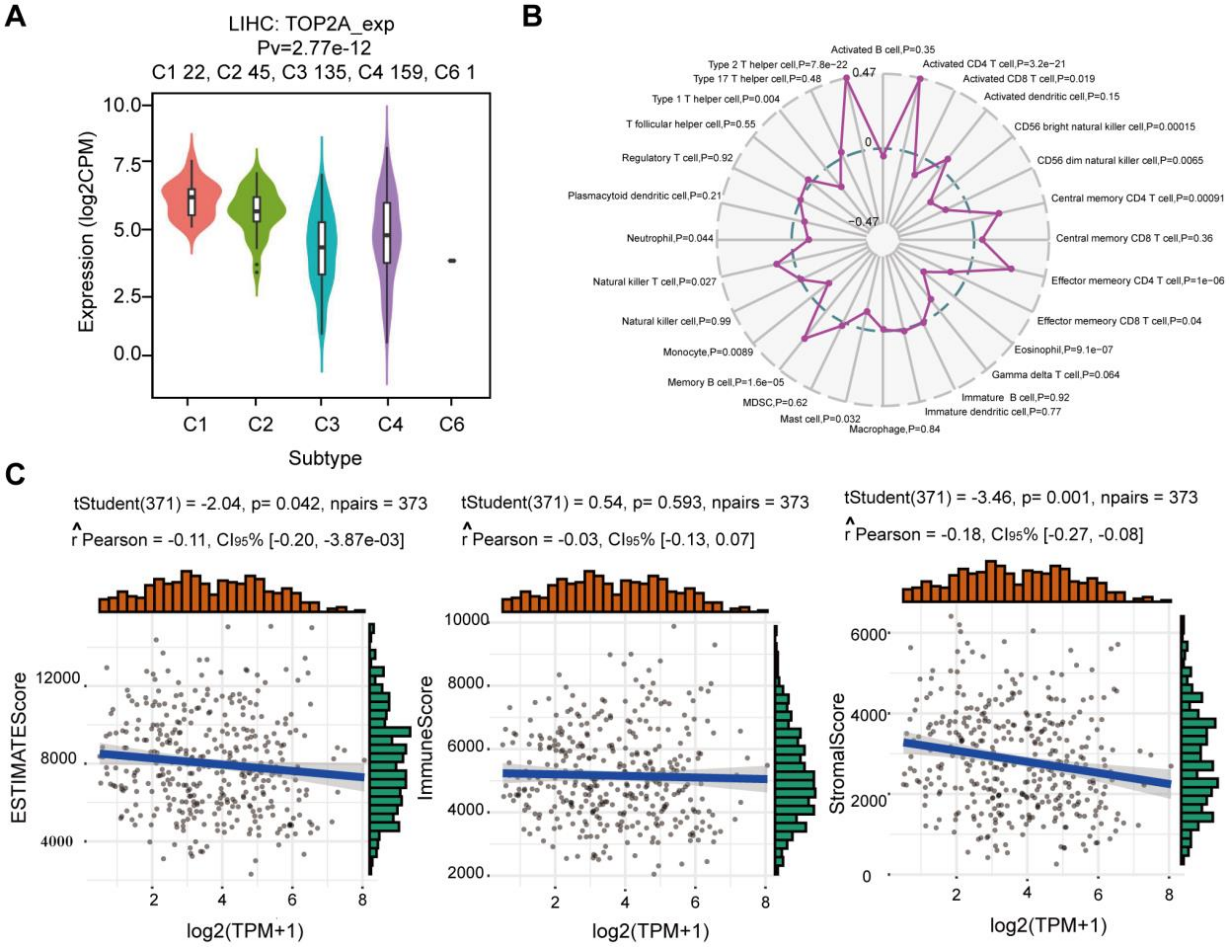
**Correlation analysis of TOP2A expression with immune infiltration and tumor microenvironment in LIHC.** (**A**) TOP2A mRNA expression in different immune subtypes in LIHC via TISIDB. (**B**) Radar map of correlation between TOP2A expression and the abundance of immune cells. (**C**) Correlation between TOP2A expression and ESTIMATE score, Immune score, and Stromal score.

**Figure 8:**
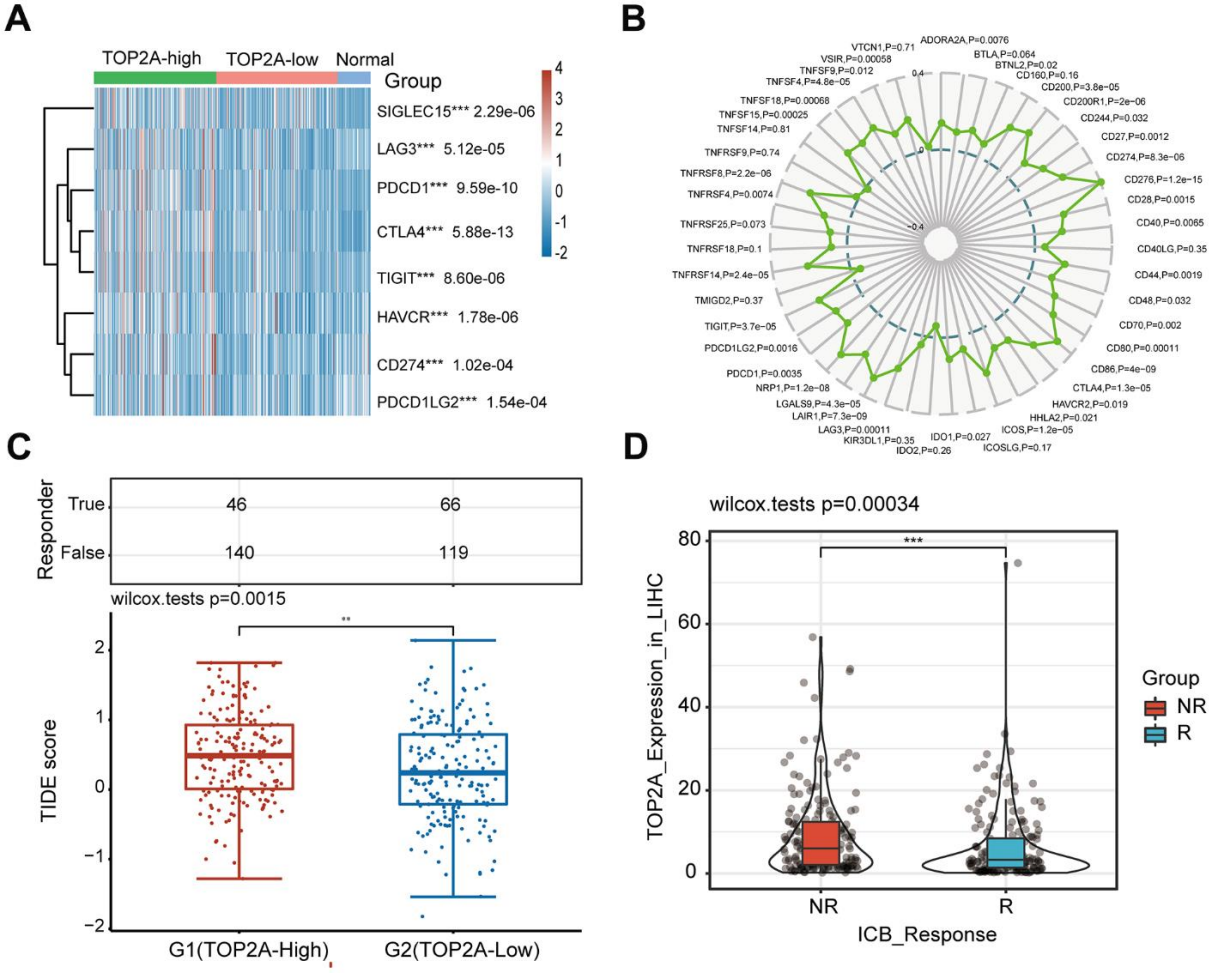
**Association analysis of TOP2A expression with immunotherapy in LIHC.** (**A**) 8 immune checkpoint genes associated with TOP2A expression. (**B**) Radar map of correlation between TOP2A expression and immune marker sets in LIHC. (**C**) Comparison of TIDE score between the higher (G1) and lower TOP2A expression (G2) groups of LIHC. (**D**) TOP2A expression in groups with a different ICB immunotherapy response status. **P < 0.01, ***P < 0.001.

Recent studies have shown that the immune microenvironment exhibits an important influence on tumor progression and prognosis [19]. Therefore, we performed a comprehensive analysis of the correlation between TOP2A expression and immune cell infiltration and response to immunotherapy in LIHC. Firstly, we studied the mRNA expression of TOP2A in different immune subtypes according to molecular typing of immune subtypes using the TISIDB database [20]. The result showed a significant difference in TOP2A expression across different immune subtypes (C1: wound healing, C2: IFN-γ dominant, C3: inflammatory, C4: lymphocyte deplete, and C6: TGF-β dominant) in LIHC (Figure 7A). The highest proportion of patients was observed in subtype C4 and the highest level of TOP2A expression was in subtype C1 in LIHC, which may suggest that TOP2A played an important role in the immune microenvironment and progress of LIHC. Correlation analysis between TOP2A expression and immune cells showed a positive relationship between TOP2A expression and the infiltration of activated CD4 T cell, central memory CD4 T cell, effector memory CD4 T cell, memory B cell, natural killer T cell, and type 2 T helper cell, while TOP2A was negatively correlated with activated CD8 T cell, CD56 bright natural killer cell, CD56 dim natural killer cell, effector memory CD8 T cell, eosinophil, mast cell, monocyte, neutrophil, and type 1 T helper cell (Figure 7B). The tumor microenvironment analysis revealed that TOP2A expression was negatively correlated with the Stromal score and ESTIMATE score, while there was no statistical significance between TOP2A expression and the Immune score (Figure 7C). Immune checkpoint inhibitor is a new tumor treatment strategy, which can effectively improve the prognosis of several malignant tumors [21]. Next, we further investigated the relationship between TOP2A expression and immune checkpoint molecules. Interestingly, Figure 8A exhibited higher expression levels of 8 immune checkpoint molecules (CD274 (PD-L1), CTLA4, HAVCR2, LAG3, PDCD1, PDCD1LG2, TIGIT, and SIGLEC15) in LIHC with higher TOP2A expression, compared with lower TOP2A expression group and normal tissue group. Moreover, the radar map demonstrated that TOP2A expression was positively correlated with most immune markers and immune checkpoint molecules, including CD274 and CTLA4 (Figure 8B). Higher TIDE scores in LIHC patients with higher TOP2A expression suggest poorer efficacy of immune checkpoint inhibition therapy (Figure 8C). Intriguingly, the expression of TOP2A was higher in the non-response ICB immunotherapy group (Figure 8D). Collectively, these results indicate that TOP2A expression may be related to tumor immune tolerance in LIHC and plays a crucial role in tumor immunotherapy and prognosis.

### Hippo-YAP signal pathway mediates TOP2A-driven LIHC cell growth, migration, and invasion

**Figure 9:**
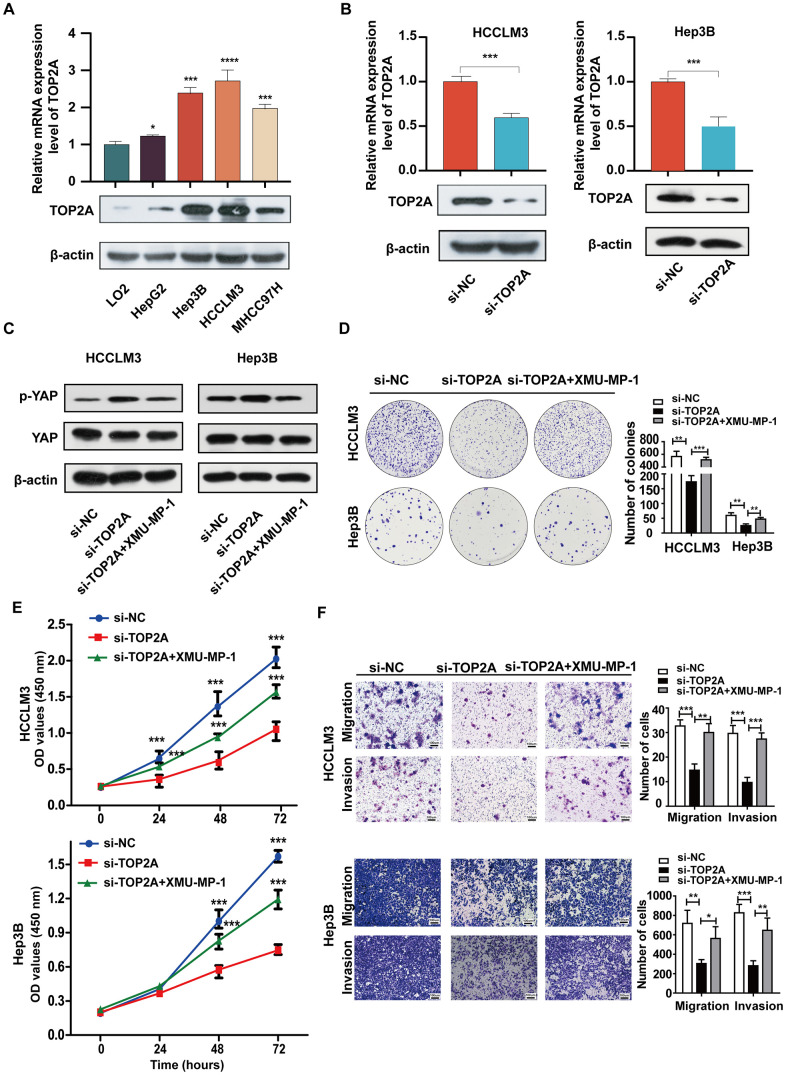
**Hippo-YAP signal pathway mediates TOP2A-driven LIHC cell growth, migration, and invasion.** (**A**) The expression of TOP2A was detected by qRT-PCR and Western blot in normal liver cell line and four liver cancer cell lines. (**B**) The knockdown efficiency of TOP2A siRNA was determined by qRT-PCR and Western blot in HCCLM3 and Hep3B cells. (**C**) The expression level of p-YAP and YAP was detected by Western blot in the indicated groups of HCCLM3 and Hep3B cells. β-actin was used as a control. (**D**, **E**) the effect of TOP2A on cell proliferation was assessed by colony formation and CCK8 assays in HCCLM3 and Hep3B cells. (**F**) The effect of the TOP2A on cell migration and invasion was tested by transwell assays in HCCLM3 and Hep3B cells. *P < 0.05, ***P < 0.001, and ****P < 0.0001. Data presented as mean ± standard deviation (SD). Experiments were repeated at least three times.

To evaluate the potential role of TOP2A in LIHC, preliminary *in vitro* experiments were performed. Firstly, we analyzed gene expression of TOP2A in a normal liver cell line (LO-2) and four liver cancer cell lines (HepG2, Hep3B, HCCLM3, and MHCC97H). The results of qRT-PCR and western blot assays indicated that TOP2A mRNA and protein levels were elevated in liver cancer cell lines compared to that in a normal liver cell line, respectively (Figure 9A and Supplementary Figure 7A), among which HCCLM3 cells with high metastatic characteristics showed the highest expression of TOP2A and Hep3B cells also exhibited higher TOP2A expression level. These findings suggested that the overexpression of TOP2A might play a critical role in tumorigenesis development. Therefore, we silenced endogenous TOP2A expression by using small interfering RNA targeting TOP2A (si-TOP2A) and conducted *in vitro* functional assays in HCCLM3 and Hep3B. The efficacy of TOP2A knockdown in HCCLM3 cells or Hep3B was tested at mRNA and protein levels (Figure 9B and Supplementary Figure 7B). As we observed that TOP2A may be associated with the Hippo-YAP signaling pathway, which was reported to be related to bone metastasis, we adopted western blot assay and verified that p-YAP protein was significantly increased when inhibiting the expression of TOP2A, and MST1/2 inhibitor XMU-MP-1 decreased the level of p-YAP in the TOP2A silencing group (Figure 9C and Supplementary Figure 7C), suggesting that TOP2A could stimulate the Hippo-YAP signaling. CCK-8 and colony formation assays were utilized to validate the effect of TOP2A knockdown on LIHC cell proliferation, revealing that the cell proliferation ability was significantly suppressed after TOP2A knockdown and could be partially rescued by XMU-MP-1 (Figure 9D, 9E). Moreover, transwell assays indicated that TOP2A knockdown could weaken the ability to migrate and invade in HCCLM3 and Hep3B cells, which could be restored by XMU-MP-1 (Figure 9F). Collectively, we conclude that TOP2A, mediating the Hippo-YAP signal pathway, could promote cancer cell growth and progression in LIHC.

### TOP2A enhances bone-specific metastatic potential and tumor-induced osteolysis in LIHC

**Figure 10:**
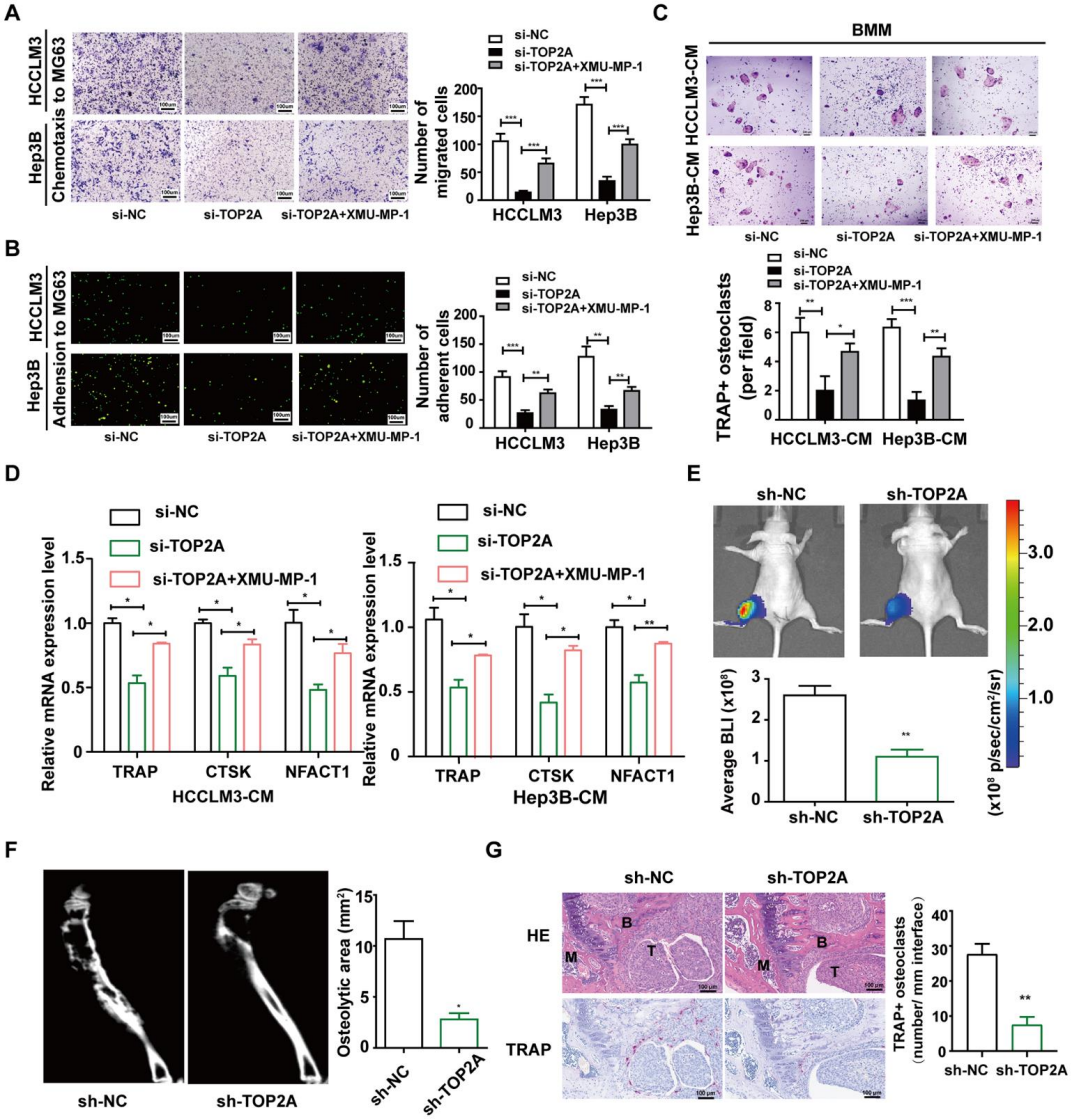
**TOP2A enhances bone-specific metastatic potential and tumor-induced osteolysis in the bone microenvironment.** (**A**) Chemotactic migration of HCCLM3 and Hep3B cells toward MG63 cells was evaluated by transwell assays. (**B**) The adhesion ability of HCCLM3 and Hep3B cells to MG63 cells was assessed by heterogeneous cell-cell adhesion assay. (**C**) TRAP staining of bone marrow monocyte (BMM) cells treated with conditioned medium (CM) from HCCLM3 and Hep3B cells. Scale bar, 200 μM. Quantification of TRAP+ osteoclast number. (**D**) qRT-PCR verified the expression of TRAP, CTSK, and NFACT1 in the indicated groups of BMM cells. GAPDH as the loading control. (**E**) 2.5×10^5^ control or TOP2A knockdown HCCLM3 and Hep3B cells were injected directly into the tibias of the mice. Representative bioluminescent images taken on day 28 after inoculation are shown. (**F**) Representative micro-CT images of tibia osteolytic lesions. (**G**) H&E and TRAP staining of tibia lesions of mice. Quantification of TRAP+ osteoclasts. Scale bar, 100 μm. *P < 0.05, **P < 0.01. B, bone; M, bone marrow; T, tumor. Data presented as mean ± SD. Experiments were repeated at least three times.

To further explore the role of TOP2A in LIHC bone metastasis, we used MG63 cells to imitate the bone microenvironment *in vitro* and detected the process of bone-specific metastasis. Through chemotactic migration and heterogeneous cell-cell adhesion assays, we revealed that knockdown of TOP2A could inhibit the chemotaxis migration ability of HCCLM3 and Hep3B cells toward MG63 cells (Figure 10A), and also heterogeneity adhesion to MG63 cells (Figure 10B), which could be partially rescued by XMU-MP-1. Moreover, since LIHC bone metastasis is typically osteolytic, involving the activation of osteoclasts, we next employed the *in vitro* osteoclastogenesis assay to confirm the functional role of TOP2A in LIHC bone metastasis. Compared to the control groups, fewer TRAP+ multi-nucleic cells were observed among mouse primary bone marrow monocyte (BMM) cells cultured with conditional medium (CM) from HCCLM3 and Hep3B cells with TOP2A silencing (Figure 10C). Simultaneously, XMU-MP-1 can restore the number of TRAP+ osteoclasts induced by the CM from TOP2A-silencing LIHC cells. qRT-PCR further showed that some osteoclast markers including TRAP, CTSK, and NFACT1 would be decreased in the BMM cells treated with CM from LIHC cells with TOP2A knockdown, while which can be rescued by XMU-MP-1(Figure 10D). These results suggested that TOP2A was involved in promoting bone-specific metastatic potential and tumor-induced osteolysis of LIHC.

Then, we further explored the effect of TOP2A *in vivo*. Four weeks after injecting control or TOP2A-knockdown HCCLM3 cells directly into the bone marrow cavity of mice tibia, a small animal bioluminescent imaging device detected that TOP2A depletion reduced tumor expansion in the tibia (Figure 10E). Besides, micro-CT of the tibia showed that mice injected with control cells had more severe osteolysis compared to mice injected with TOP2A-knockdown HCCLM3 cells (Figure 10F). Histological H&E staining confirmed a decrease in tibia tumor burden in mice treated with TOP2A-knockdown HCCLM3 cells. TRAP staining found that TOP2A knockdown decreased the number of TRAP+ osteoclasts at the bone-tumor interface (Figure 10G). Therefore, these findings demonstrate that TOP2A is responsible for osteolytic metastasis and tumor-induced osteolysis in LIHC.

## DISCUSSION

The incidence of liver hepatocellular carcinoma is rising globally and could reach 1 million cases per year in the next decade [22]. At present, the morbidity of LIHC is fourth, and the mortality rate is second in China, which is higher than the world average level [23]. With the application of targeted therapy and immunotherapy, the survival time of advanced LIHC has been significantly prolonged, and the median survival time can reach 19 months [24, 25]. With the discovery of more patients, studies found that bone metastasis is an independent risk factor for the prognosis of LIHC, and skeletal-related events seriously affect the quality of life for LIHC patients [26]. Therefore, the early diagnosis and treatment for patients with LIHC bone metastasis are urgent. The in-depth study of molecular mechanisms is the research focus that needs to be solved in the future. Although some studies have reported the molecular mechanism of bone metastasis of LIHC, there are still many challenges to break through [27, 28].

This study identified TOP2A as a gene related to bone metastasis of LIHC and analyzed its potential as a prognostic biomarker through a series of bioinformatics studies. Surprisingly, TOP2A was downregulated in the liver tumors from LIHC-BM patients, while displaying a higher level in those from non-BM patients by analyzing the mRNA expression data of GSE27635 dataset. For this, two reasons were speculated: on the one hand, the relatively limited sample size and large tumor heterogeneity make it impossible to fully represent the actual situation. On the other hand, the current sequencing data comes from the primary liver tumors of LIHC patients. Concerning the progress of tumor bone metastasis, tumor cells from the primary liver site to the bone marrow niches, tumor cells would develop specific molecular traits to better fit in the bone microenvironment, and we speculated that TOP2A may be highly expressed in the tumor cells with stronger BM preferences. Moreover, a series of experiments were conducted to verify the relationship of TOP2A with LIHC bone metastasis. qRT-PCR and IHC assay demonstrated that both the mRNA and protein levels of TOP2A were elevated in LIHC-BM lesions compared with the LIHC tumor tissues. More importantly, *in vitro* and *in vivo* experiments confirmed that TOP2A was responsible for bone-specific metastatic and tumor-induced osteolysis in LIHC.

Notably, our study further analyzed the expression and prognostic value of TOP2A in tumors. Pan-cancer analysis of the TCGA database confirmed that the abnormally high expression of TOP2A in a variety of tumors was related to the poor prognosis of LIHC patients, uncovering the role of TOP2A as an oncogene. Importantly, the abnormal expression of TOP2A was positively correlated with metastasis. Our findings are consistent with many previously reported results. For example, as a key oncogene in the prostate, TOP2A is highly expressed in patients with metastatic prostate, which can be used as a biomarker for early diagnosis and treatment of prostate cancer [29]. TOP2A facilitates the progression of lung adenocarcinoma cells and predicts the adverse prognosis of lung adenocarcinoma [30]. Moreover, multiple databases of TCGA, ICGC, and GEO further verified that TOP2A was highly expressed in LIHC and was associated with poor prognosis, which was in keeping with recent studies [31]. We further found that the high expression of TOP2A in LIHC was related to DNA hypomethylation and p53 mutation, which provided new ideas for the pathogenesis of TOP2A.

We further explored the potential functions and molecular mechanisms of TOP2A in LIHC. Enrichment analysis showed that TOP2A expression was related to cell cycle, DNA replication, DNA damage repair, chromosome modification, and metastasis, suggesting that it may play an important role in the occurrence and development of tumors. It is worth noting that TOP2A was not only positively correlated with PI3K / AKT/ mTOR tumor signal pathway but also correlated with metastasis-related EMT, MMP protein, angiogenesis, and the Hippo-YAP pathway. Interestingly, recent studies have confirmed that the Hippo-YAP signal transduction pathway plays an important role in carcinogenesis, tissue regeneration, and functional regulation of stem cells [32, 33]. At the same time, it plays a key role in regulating the balance between osteoblast bone formation and osteoclast bone resorption and maintaining bone homeostasis [34]. ROR1-HER3-LncRNA signal axis could regulate tumor bone metastasis by regulating the Hippo-YAP signal pathway [35]. ABL kinase could regulate tumor bone interaction through TAZ (Hippo pathway mediator) and STAT5 to promote osteolytic metastasis of breast cancer [36]. We conducted preliminary *in vitro* and *in vivo* experiments to confirm that TOP2A, mediating the Hippo-YAP signal pathway, could promote cancer cell growth, metastasis, and osteoclastogenesis in LIHC. The process of bone metastasis includes cancer cell growth, migration, invasion, homing to and residing in bone microenvironment, and osteoclastogenesis, therefore, TOP2A may be used as a new potential target for bone metastasis of LIHC.

In recent years, immunotherapy has gradually become the focus of basic and clinical research. As we all know, changes in the immune microenvironment may play an important role in immune resistance and immune escape, leading to the recurrence and metastasis of LIHC [37]. In recent years, immunotherapy represented by immune checkpoint inhibitors has brought new hope to patients with advanced LIHC [38, 39]. Our study found that TOP2A was lowly expressed in the C3 immune subtype and highly expressed in the C1 immune subtype, suggesting that high expression of TOP2A is related to immunosuppression and poor prognosis. Further analysis showed that the expression of TOP2A was negatively correlated with activated CD8 T cells and positively correlated with immune checkpoint molecules such as CD274 (PD-L1) and CTLA4, which suggested that high expression of TOP2A was related to immune tolerance in LIHC. Meanwhile, TOP2A was negatively correlated with the Stromal score and ESTIMATE score, which also suggested that the high expression of TOP2A was positively correlated with tumor purity and poor prognosis. Further analysis of immune checkpoint treatment response showed that high TOP2A expression predicted the poor treatment effect of immune checkpoint inhibitors. Overall, our study found that TOP2A overexpression may lead to immune escape and immunotherapy failure, resulting in tumor progression and poor overall prognosis of LIHC.

Precisely targeted therapy for patients with advanced LIHC will be the main direction in the future. This study preliminarily identified TOP2A as a potential target for LIHC bone metastasis. However, this study still has some limitations. First, more samples and independent cohorts are needed for more precise and robust analyses. Second, more in-depth molecular experiments are demanded to explore the role and mechanism of TOP2A in LIHC bone metastasis, and prospective studies are required to confirm its clinical translation potential.

## CONCLUSIONS

In conclusion, we identified TOP2A as a gene related to bone metastasis of LIHC and explored its relationship with poor prognosis and immunosuppressive tumor microenvironment of LIHC through a comprehensive analysis of bioinformatics. Enrichment analysis and preliminary *in vitro* and *in vivo* experiments confirmed that TOP2A, mediating the Hippo-YAP signal pathway, could promote cancer cell growth, bone-specific metastatic potential and tumor-induced osteolysis in LIHC. This study provides novel insights into the pathogenesis of LIHC bone metastasis, and TOP2A may serve as a new potential therapeutic target and prognostic biomarker of bone metastasis of LIHC.

## MATERIALS AND METHODS

### Data source and acquisition

The mRNA expression data and clinical information of 48 LIHC patients with and without BM, were obtained from Gene Expression Omnibus (GEO) database (GSE27635) [40]. The other ten public LIHC microarray datasets were also extracted from the GEO database (http://www.ncbi.nlm.nih.gov/geo), including GSE22058, GSE25097, GSE36376, GSE14520, GSE10143, GSE46444, GSE54236, GSE63898, GSE64041, GSE76427. The mRNA data and corresponding clinical information of pan-cancer were derived from the UCSC Xena database (http://xena.ucsc.edu/) [41]. RNA sequencing data of LIHC were acquired from the TCGA database (https://portal.gdc.cancer.gov/), which included gene expression, genomic mutation, DNA methylation, and clinical data [42]. The RNA expression information of normal liver tissue came from Genotype-Tissue Expression (GTEx) database (https://commonfund.nih.gov/GTEx/) [43]. Transcriptome RNA-seq data and clinical follow-up data of LIHC were retrieved from the ICGC database (https://dcc.icgc.org/) [44]. The cell lines mRNA expression of LIHC was derived from the CCLE dataset (https://portals.broadinstitute.org/ccle/about) [45].

### Screening of differentially expressed genes (DEGs) associated with bone metastasis of LIHC

After preprocessing RNA-seq data of LIHC from the GSE27635 dataset, the R package “Limma” was used to screen DEGs between LIHC with BM and without BM, or LIHC and paracancerous normal tissue. **|**log2 fold change (FC)**|** >1.5 and *P*-Value <0.05 were regarded as the thresholds to identify DEGs. The results were presented with volcano plot and heatmap. The intersection of DEGs was obtained by using the “VennDiagram” package. The overlapping BM-related genes were further subjected to functional enrichment analyses.

### Analysis of TOP2A expression in pan-cancer and LIHC

The RNA expression levels of TOP2A in pan-cancer or LIHC and corresponding normal tissues were obtained from the TCGA/GTEx/GEO/ICGC database. The R v4.0.3 software and package ggplot2 (v3.3.3) were used for the above analyses and visualization. Correlations between TOP2A expression and clinical characteristics of LIHC were investigated using the TCGA database. The protein expression levels of TOP2A in LIHC and normal liver tissues were evaluated using the Human Protein Atlas (HPA) database (http://www.proteinatlas.org/), which is an open-access resource to search for specific genes/proteins for human proteins [46]. The images were available from v23.proteinatlas.org via https://www.proteinatlas.org/ENSG00000131747-TOP2A/tissue/liver#img and https://www.proteinatlas.org/ENSG00000131747-TOP2A/pathology/liver+cancer#img.

### Prognosis analysis

The survival data of pan-cancer were acquired from the TCGA database. The “survival” and “survminer” R packages were used to construct the Kaplan–Meier curves with log-rank test, and the forest maps with *P*-value, HR, and 95% CI were constructed by the R “forestplot” package. HR > 1 and log-rank *P* < 0.05 were considered to be a risk factor for prognosis and statistically significant. The multivariable Cox proportional hazard model was conducted using the TIMER database (https://cistrome.shinyapps.io/timer/) to further explore whether TOP2A was an independent factor in LIHC patients. The receiver operating characteristic (ROC) curves for 1-, 2-, and 3-year were generated with R package “timeROC” to evaluate the survival predictive power, and areas under the curve (AUC) were calculated.

### Analysis of DNA methylation and genomic alterations

For DNA methylation analysis, the DNA methylation data were downloaded from the TCGA database. The R package “methylKit” was used to assess DNA methylation levels in LIHC and normal liver tissues. Correlation between DNA methylation level and TOP2A RNA expression, and survival of LIHC were further explored with corresponding R package. The somatic mutation data of LIHC from TCGA were visualized using the R package “maftools”. Mutant types included missense mutation, in frame del, nonsense mutation, splice site, in frame ins, frame shift del, frame shift ins, and multi hit. The R package was applied to further explore the relationship between TP53 mutation and TOP2A expression, and survival of LIHC.

### Functional enrichment analysis

According to the median value of TOP2A expression, they were divided into TOP2A high and low expression groups in LIHC. **|**log2 fold change (FC)**|** >2 and *P*-Value <0.05 were regarded as the thresholds to identify DEGs. The heatmap constructed by the “pheatmap” R package showed the top 50 co-expressed genes with TOP2A in LIHC. Gene Ontology (GO) and Kyoto Encyclopedia of Genes and Genomes (KEGG) analysis were performed with the R package “Cluster Profiler” to analyze the potential function and molecular mechanism of TOP2A in LIHC. The R package “ggplot2” was used to perform gene set enrichment analysis (GSEA) analysis. The spearman correlations between TOP2A expression of LIHC and pathways or biomarkers related to metastasis or proliferation were analyzed with R software GSVA package or TIMER web server (https://cistrome.shinyapps.io/timer/) [47].

### Analysis of immune infiltration and immunotherapy

TOP2A gene expression across diverse immune subtypes in LIHC was analyzed through TISIDB (http://cis.hku.hk/TISIDB/), which is a web portal for tumor and immune system interaction [48]. The immune subtypes included C1 (wound healing), C2 (IFN-γ dominant), C3 (inflammatory), C4 (lymphocyte deplete), and C6 (TGF-β dominant) subtypes. The “fmsb” R package was utilized to analyze the relationship between the expression of TOP2A and the abundance of tumor immune cell infiltration. ESTIMATE provides researchers with scores for tumor purity, the level of stromal cells, and the infiltration level of immune cells in tumor tissues based on expression data (https://bioinformatics.mdanderson.org/estimate/index.html), which was used to evaluate the relationships between the expression of TOP2A and ESTIMATE score, immune score, and stromal score. The heatmap was applied to analyze the expression distribution of immune checkpoints gene in tumor tissues and normal tissues using the “ggplot2” R package and “pheatmap” R package. The relationship between TOP2A expression and immune markers set was assessed with CIBERSORT algorithm and visualized with “fmsb” R package. Potential immune checkpoint blockade (ICB) response was predicted with Tumor Immune Dysfunction and Exclusion (TIDE) (http://cis.hku.hk/TISIDB/index.php) and ImmuCellAI (http://bioinfo.life.hust.edu.cn/ImmuCellAI#!/) algorithm.

### Patient samples

The Fresh tissue samples of 34 normal liver, 36 LIHC, and 13 liver cancer bone metastasis lesions were obtained at Tianjin Medical University Cancer Institute and Hospital. These tissues were used to detect the RNA expression level of TOP2A. The tissue microarray was purchased from Xi’an Biotech Co., Ltd (cat.no. D950601, Xi’an, China), consisting of 79 LIHC tissues and 16 liver tissues. Among them, 79 cases of LIHC included 42 cases in TNM stage II, 30 cases in stage III, and 2 cases in stage IV, others were unknown. 3 paired primary LIHC and counterpart LIHC bone metastasis sections were collected from Tianjin Medical University Cancer Institute and Hospital. The tissue microarray and sections were used to detect protein expression levels of TOP2A.

### Immunohistochemistry (IHC)

The expression of TOP2A protein in paraffin-embedded tissue from patients was evaluated using IHC. In short, 4-μm tissue slices were sequentially deparaffinized by xylene and rehydrated in gradient ethanol, followed by antigen repair with citrate antigen repair buffer (PH6.0). The sections were then exposed to 3% hydrogen peroxide solution to block endogenous peroxidase activity. After blocking with serum for 30 minutes at room temperature, the slices were incubated with primary antibodies against TOP2A (dilution 1:2000; cat no. ab52934, Abcam, USA) overnight at 4° C. Next day, the sections were incubated with secondary antibodies at room temperature for 1 h, and then stained with 3,3’ -diaminobenzidine (ZSGB bio, Beijing, China). The IHC score of protein expression was calculated by multiplying the intensity score and the percentage score. The staining intensity score was designated as follows: 0 (no staining), 1 (light yellow), 2 (brown) and 3 (brownish red). The percentage score was graded as follows: 0 (no positive), 1 (positive<10%), 2 (10%≤positive<50%), 3 (50%≤positive<80%) and 4 (positive≥80%).

### Cell culture, transfection, and treatment

The normal human liver cell line LO2 and four liver cancer cell lines (HepG2, Hep3B, MHCC97H, and HCCLM3) were obtained from Professor Zhang Xiaodong. HEK-293T cell lines were purchased from American Type Culture Collection (ATCC, Manassas, VA, USA). Four liver cancer cell lines and HEK-293T cells were cultured in Dulbecco’s modified Eagle’s medium (DMEM; Cellmax, Beijing, China) containing 10% fetal bovine serum (FBS; Cellmax, Beijing, China) at 37° C in an environment with 5% CO2. LO2 was maintained in 1640 medium containing 10% FBS. Small interfering RNA (siRNA) and small hairpin RNA (shRNA) targeting TOP2A and corresponding negative control siRNA and shRNA were purchased from Shenggong (Shanghai, China). The sequence of TOP2A siRNA was as follows: CCCAACTTTGATGTGCGTGAA. We used a transfection reagent (Zeta Life, Menlo Park，CA, USA) to transfect siRNA according to the manufacturer’s instructions. After transfection 48 h, cells were harvested for RNA and protein extraction and processed for functional assays. For *in vivo* knockdown of TOP2A in xenograft experiments, the shRNA sequence was cloned into pLKO.1 lentiviral vector and used to construct a stable knockdown TOP2A HCCLM3 cell line. For rescue experiments, TOP2A silencing cells, including HCCLM3 and Hep3B, were treated with the MST1/2 inhibitor XMU-MP-1 (3 μM, MedChemExpress, Shanghai, China) for 24 h.

### qRT-PCR

Total RNA was isolated using TRIzol reagent (Yeasen, Shanghai, China), and cDNA was synthesized using the cDNA Synthesis Kit (TransGen, China). Relative quantification of gene expression level was conducted using FastStart Universal SYBR Green Master Mix (Yeasen, China) with GAPDH mRNA as an endogenous control. The expression values of target genes were calculated using the 2 ^−ΔΔCT^ method. The sequences of gene-specific primers were as follows: GAPDH (human) forward primer, 5’-CATGTACGTTGCTATCCAGGC-3’; GAPDH (human) reverse primer, 5’-CTCCTTAATGTCACGCACGAT-3’. TOP2A (human) forward primer, 5’- ACCATTGCAGCCTGTAAATGA-3’; TOP2A (human) reverse primer, 5’- GGGCGGAGCAAAATATGTTCC-3’. TRAP (mouse) forward primer, 5’- GCAACATCCCCTGGTATGTG-3’; TRAP (mouse) reverse primer, 5’- GCAAACGGTAGTAAGGGCTG-3’. CTSK (mouse) forward primer, 5’- ATGTGAACCATGCAGTGTTGGTGG-3’; CTSK (mouse) reverse primer, 5’- ATGCCGCAGGCGTTGTTCTTATTC-3’. NFATC1 (mouse) forward primer, 5’- CAGTGTGACCGAAGATACCTGG-3’; NFATC1 (mouse) reverse primer, 5’- TCGAGACTTGATAGGGACCCC-3’. GAPDH (mouse) forward primer, 5’- TGTTTCCTCGTCCCGTAG-3’; GAPDH (mouse) reverse primer, 5’- CAATCTCCACTTTGCCACT -3’.

### Western blot

Total protein was isolated from cells using RIPA reagent (Solarbio, Beijing, China) containing 10% PMSF inhibitor. Protein samples were separated using 10% SDS-PAGE for 2 h and transferred into PVDF membranes at 250 mA for 1.5 h. After blocking with 10% non-fat milk for 2 h, primary antibodies against TOP2A (dilution 1:1000; cat no.ab52934, Abcam, USA), YAP (dilution 1:1000; cat no.ab52771, Abcam, USA), p-YAP (dilution 1:1000; cat no. ab76252, Abcam, USA) and β-actin (dilution 1:12000; cat no. AF7018, Affinity, China) were incubated at 4° C overnight. The membranes were then incubated with secondary antibodies after washing three times with TBST. Finally, the ECL reagent was used to visualize the bands, and β-actin was used as a loading control to calculate the relative expression of target proteins.

### Cell count kit-8 (CCK-8) assay

A total of 3×10^3^ HCCLM3 or Hep3B cells were plated into the 96-well plate. After 24 h, 48 h, and 72 h, 10 ul of CCK-8 reagent (Yeasen, Shanghai, China) was added to each well. After incubation for 1 h at 37° C, the absorbance of each well at 450 nm was detected.

### Transwell assay

The cell migration and invasion ability were evaluated using the transwell assay. 5 x 10^5 HCCLM3 or 1 x 10^5 Hep3B cells suspended in 200 μL serum-free DMEM were added to each transwell cell culture insert (LABSELECT, China; pore size: 8 μm), whereas 600 μL DMEM containing 20% FBS was placed in the lower chamber in 24-well dishes. After incubation for 24 h at 37° C, migrated or invasive cells through the membrane were fixed with methanol for 10 min and stained with 0.1% crystal violet for 30 min. After staining, cells were calculated and imaged under the microscope.

### Colony formation assays

Transfected cells were seeded in 6 well plates at a density of 500 cells/well and then cultured for 14 days. The formed colonies were fixed with methanol and then stained with 0.1% crystal violet.

### Chemotactic migration assay

*In vitro* Chemotactic migration assay of HCCLM3 or Hep3B cells toward a mimic bone microenvironment was performed using transwell cell culture inserts. Firstly, the osteogenic MG63 cells were pre-seeded into the bottom chambers of the 24-well plate. Then, 5 x 10^5 HCCLM3 or 1 x 10^5 Hep3B cells suspended in 200 μL serum-free DMEM were added to the upper chambers when MG63 cells reached about 80% confluent. After 24 h, the migrating cells were fixed with methanol, stained with 0.1% crystal violet, and counted under a microscope.

### Heterogeneous cell-cell adhesion assay

1 x 10^5 HCCLM3 or Hep3B cells labeled with Green fluorescent protein (GFP) were inoculated into 24-well plate pre-inoculated with MG63 at a confluence of nearly 100%. After 30 minutes, non-adherent cancer cells were washed away by PBS and counted under a microscope.

### Osteoclastogenesis assay *in vitro*

Primary BMM cells were isolated from femur and tibia of 6-week-old C57BL/6 mice and cultured in basal culture medium (α-MEM supplemented with 10% FBS and 1% penicillin/streptomycin) overnight. The next day, non-adherent cells were plated in 24-well plates at 1×105 per well in a 4:1 mixture of basal culture medium/filtered conditional medium (collected from Hep3B and HCCLM3 cells) supplemented with recombinant murine M-CSF (40 ng/mL, Amizona Scientific, LLC, Birmingham, AL, USA). The day 3, recombinant murine sRANKL (50 ng/mL, Amizona Scientific, LLC, Birmingham, AL, USA) was added and replaced medium every 3 days. The tartrate-resistant acid phosphatase (TRAP) staining was conducted on day 7 according to the manufacturer’s instructions (Amizona Scientific, LLC, Birmingham, AL, USA). TRAP+ multinucleated osteoclasts (multinucleated cells ≥3 nuclei) were quantified as mature osteoclasts and counted under a microscope for analysis. qRT-PCR examined the expression of TRAP, CTSK, and NFACT1 (osteoclast markers) in the above BMM.

### Animal experiments

Female BALB/c-nude mice (4-6 weeks old) were purchased from Beijing SPF Biotechnology Co., Ltd (China) and maintained under a specific-pathogen free (SPF) facility. All animal procedures were approved by the Animal Care Committee of the Tianjin Medical University Cancer Institute and Hospital and handled in accordance with the Guide for the Care and Use of Laboratory Animals. For intra-tibia injections, mice were anesthetized using isoflurane inhalation. HCCLM3-luc-sh-NC cells and HCCLM3-luc-sh-TOP2A cells (2.5 × 10^5^ in 25 ul PBS) were injected into the tibia bone marrow cavity of 5 mice in each group respectively. Bioluminescence imaging acquired with the IVIS system (Caliper Life Sciences, Hopkinton, MA, USA) was used to detect cancer cell inoculation and progression. The osteolytic lesions caused by the tumor on the tibia were visualized using micro-CT imaging (Inviscan IRIS, Strasbourg, France). After 4 weeks, the hindlimb bones were excised, fixed in 10% neutral-buffered formalin, decalcified with 10% EDTA for 3 weeks, embedded in paraffin for hematoxylin and subjected to eosin (H&E) and TRAP staining. Mature osteoclast number was identified by multinucleated TRAP+ cells along the tumor–bone interface on TRAP-stained sections and quantified as osteoclast number per millimeter interface.

### Statistical analysis

All bioinformatics statistical analyses and R packages were performed using R software version 4.0.3. The experimental data were presented as Mean ± SD. Student’s t-test and one-way ANOVA were used to evaluate the difference between groups. Each assay should be conducted independently at least three times. All statistical analyses of experiments were performed with GraphPad Prism 5.0. Unless otherwise specified, a *P*-value <0.05 was considered statistically significant.

## Supplementary Material

Supplementary Figures

## References

[1] Sung H, Ferlay J, Siegel RL, Laversanne M, Soerjomataram I, Jemal A, Bray F. Global Cancer Statistics 2020: GLOBOCAN Estimates of Incidence and Mortality Worldwide for 36 Cancers in 185 Countries. CA Cancer J Clin. 2021; 71:209–49. 10.3322/caac.21660 33538338

[2] Llovet JM, Kelley RK, Villanueva A, Singal AG, Pikarsky E, Roayaie S, Lencioni R, Koike K, Zucman-Rossi J, Finn RS. Hepatocellular carcinoma. Nat Rev Dis Primers. 2021; 7:6. 10.1038/s41572-020-00240-3 33479224 PMC13537433

[3] Zhang X, El-Serag HB, Thrift AP. Predictors of five-year survival among patients with hepatocellular carcinoma in the United States: an analysis of SEER-Medicare. Cancer Causes Control. 2021; 32:317–25. 10.1007/s10552-020-01386-x 33394207

[4] Liang JY, Wang DS, Lin HC, Chen XX, Yang H, Zheng Y, Li YH. A Novel Ferroptosis-related Gene Signature for Overall Survival Prediction in Patients with Hepatocellular Carcinoma. Int J Biol Sci. 2020; 16:2430–41. 10.7150/ijbs.45050 32760210 PMC7378635

[5] Zhang L, Niu H, Yang P, Ma J, Yuan BY, Zeng ZC, Xiang ZL. Serum lnc34a is a potential prediction biomarker for bone metastasis in hepatocellular carcinoma patients. BMC Cancer. 2021; 21:161. 10.1186/s12885-021-07808-6 33588789 PMC7885499

[6] Harding JJ, Abu-Zeinah G, Chou JF, Owen DH, Ly M, Lowery MA, Capanu M, Do R, Kemeny NE, O’Reilly EM, Saltz LB, Abou-Alfa GK. Frequency, Morbidity, and Mortality of Bone Metastases in Advanced Hepatocellular Carcinoma. J Natl Compr Canc Netw. 2018; 16:50–8. 10.6004/jnccn.2017.7024 29295881

[7] Huang Z, Chu L, Liang J, Tan X, Wang Y, Wen J, Chen J, Wu Y, Liu S, Liao J, Hou R, Ding Z, Zhang Z, et al. H19 Promotes HCC Bone Metastasis Through Reducing Osteoprotegerin Expression in a Protein Phosphatase 1 Catalytic Subunit Alpha/p38 Mitogen-Activated Protein Kinase-Dependent Manner and Sponging microRNA 200b-3p. Hepatology. 2021; 74:214–32. 10.1002/hep.31673 33615520

[8] Nielsen CF, Zhang T, Barisic M, Kalitsis P, Hudson DF. Topoisomerase IIα is essential for maintenance of mitotic chromosome structure. Proc Natl Acad Sci USA. 2020; 117:12131–42. 10.1073/pnas.2001760117 32414923 PMC7275761

[9] Ma W, Wang B, Zhang Y, Wang Z, Niu D, Chen S, Zhang Z, Shen N, Han W, Zhang X, Wei R, Wang C. Prognostic significance of TOP2A in non-small cell lung cancer revealed by bioinformatic analysis. Cancer Cell Int. 2019; 19:239. 10.1186/s12935-019-0956-1 31528121 PMC6737627

[10] Boot A, Liu M, Stantial N, Shah V, Yu W, Nitiss KC, Nitiss JL, Jinks-Robertson S, Rozen SG. Recurrent mutations in topoisomerase IIα cause a previously undescribed mutator phenotype in human cancers. Proc Natl Acad Sci USA. 2022; 119:e2114024119. 10.1073/pnas.2114024119 35058360 PMC8795545

[11] Jiang YZ, Liu Y, Xiao Y, Hu X, Jiang L, Zuo WJ, Ma D, Ding J, Zhu X, Zou J, Verschraegen C, Stover DG, Kaklamani V, et al. Molecular subtyping and genomic profiling expand precision medicine in refractory metastatic triple-negative breast cancer: the FUTURE trial. Cell Res. 2021; 31:178–86. 10.1038/s41422-020-0375-9 32719455 PMC8027015

[12] Wang B, Shen Y, Zou Y, Qi Z, Huang G, Xia S, Gao R, Li F, Huang Z. TOP2A Promotes Cell Migration, Invasion and Epithelial-Mesenchymal Transition in Cervical Cancer via Activating the PI3K/AKT Signaling. Cancer Manag Res. 2020; 12:3807–14. 10.2147/CMAR.S240577 32547216 PMC7251484

[13] Zhang F, Wu H. MiR-599 targeting TOP2A inhibits the malignancy of bladder cancer cells. Biochem Biophys Res Commun. 2021; 570:154–61. 10.1016/j.bbrc.2021.06.069 34284141

[14] Grenda A, Błach J, Szczyrek M, Krawczyk P, Nicoś M, Kuźnar Kamińska B, Jakimiec M, Balicka G, Chmielewska I, Batura-Gabryel H, Sawicki M, Milanowski J. Promoter polymorphisms of TOP2A and ERCC1 genes as predictive factors for chemotherapy in non-small cell lung cancer patients. Cancer Med. 2020; 9:605–14. 10.1002/cam4.2743 31797573 PMC6970032

[15] Tian D, Yu Y, Zhang L, Sun J, Jiang W. A Five-Gene-Based Prognostic Signature for Hepatocellular Carcinoma. Front Med (Lausanne). 2021; 8:681388. 10.3389/fmed.2021.681388 34568357 PMC8455941

[16] Zhuo W, Kang Y. Lnc-ing ROR1-HER3 and Hippo signalling in metastasis. Nat Cell Biol. 2017; 19:81–3. 10.1038/ncb3467 28139652

[17] Lee J, Youn BU, Kim K, Kim JH, Lee DH, Seong S, Kim I, Han SH, Che X, Choi JY, Park YW, Kook H, Kim KK, Lim DS, Kim N. Mst2 Controls Bone Homeostasis by Regulating Osteoclast and Osteoblast Differentiation. J Bone Miner Res. 2015; 30:1597–607. 10.1002/jbmr.2503 25761670

[18] Zhao H, Hu H, Chen B, Xu W, Zhao J, Huang C, Xing Y, Lv H, Nie C, Wang J, He Y, Wang SQ, Chen XB. Overview on the Role of E-Cadherin in Gastric Cancer: Dysregulation and Clinical Implications. Front Mol Biosci. 2021; 8:689139. 10.3389/fmolb.2021.689139 34422902 PMC8371966

[19] Meng J, Lu X, Zhou Y, Zhang M, Gao L, Gao S, Yan F, Liang C. Characterization of the prognostic values and response to immunotherapy/chemotherapy of Krüppel-like factors in prostate cancer. J Cell Mol Med. 2020; 24:5797–810. 10.1111/jcmm.15242 32281273 PMC7214179

[20] Thorsson V, Gibbs DL, Brown SD, Wolf D, Bortone DS, Ou Yang TH, Porta-Pardo E, Gao GF, Plaisier CL, Eddy JA, Ziv E, Culhane AC, Paull EO, et al, and Cancer Genome Atlas Research Network. The Immune Landscape of Cancer. Immunity. 2018; 48:812–30.e14. 10.1016/j.immuni.2018.03.023 29628290 PMC5982584

[21] Topalian SL, Drake CG, Pardoll DM. Immune checkpoint blockade: a common denominator approach to cancer therapy. Cancer Cell. 2015; 27:450–61. 10.1016/j.ccell.2015.03.001 25858804 PMC4400238

[22] Llovet JM, Montal R, Sia D, Finn RS. Molecular therapies and precision medicine for hepatocellular carcinoma. Nat Rev Clin Oncol. 2018; 15:599–616. 10.1038/s41571-018-0073-4 30061739 PMC12452113

[23] Zheng R, Zhang S, Zeng H, Wang S, Sun K, Chen R, Li L, Wei W, He J. Cancer incidence and mortality in China, 2016. Journal of the National Cancer Center. 2022; 2:1–9. 10.1016/j.jncc.2022.02.002 PMC11256658 39035212

[24] Llovet JM, Castet F, Heikenwalder M, Maini MK, Mazzaferro V, Pinato DJ, Pikarsky E, Zhu AX, Finn RS. Immunotherapies for hepatocellular carcinoma. Nat Rev Clin Oncol. 2022; 19:151–72. 10.1038/s41571-021-00573-2 34764464

[25] Finn RS, Qin S, Ikeda M, Galle PR, Ducreux M, Kim TY, Kudo M, Breder V, Merle P, Kaseb AO, Li D, Verret W, Xu DZ, et al, and IMbrave150 Investigators. Atezolizumab plus Bevacizumab in Unresectable Hepatocellular Carcinoma. N Engl J Med. 2020; 382:1894–905. 10.1056/NEJMoa1915745 32402160

[26] Zhang S, Xu Y, Xie C, Ren L, Wu G, Yang M, Wu X, Tang M, Hu Y, Li Z, Yu R, Liao X, Mo S, et al. RNF219/*α*-Catenin/LGALS3 Axis Promotes Hepatocellular Carcinoma Bone Metastasis and Associated Skeletal Complications. Adv Sci (Weinh). 2020; 8:2001961. 10.1002/advs.202001961 Erratum in: Adv Sci (Weinh). 2021; 8:e2102956. 10.1002/advs.202001961 33643786 PMC7887580

[27] Sun C, Hu A, Wang S, Tian B, Jiang L, Liang Y, Wang H, Dong J. ADAM17-regulated CX3CL1 expression produced by bone marrow endothelial cells promotes spinal metastasis from hepatocellular carcinoma. Int J Oncol. 2020; 57:249–63. 10.3892/ijo.2020.5045 32319605 PMC7252465

[28] Zhang L, Niu H, Ma J, Yuan BY, Chen YH, Zhuang Y, Chen GW, Zeng ZC, Xiang ZL. The molecular mechanism of LncRNA34a-mediated regulation of bone metastasis in hepatocellular carcinoma. Mol Cancer. 2019; 18:120. 10.1186/s12943-019-1044-9 31349837 PMC6659280

[29] Labbé DP, Sweeney CJ, Brown M, Galbo P, Rosario S, Wadosky KM, Ku SY, Sjöström M, Alshalalfa M, Erho N, Davicioni E, Karnes RJ, Schaeffer EM, et al. TOP2A and EZH2 Provide Early Detection of an Aggressive Prostate Cancer Subgroup. Clin Cancer Res. 2017; 23:7072–83. 10.1158/1078-0432.CCR-17-0413 28899973 PMC5690819

[30] Kou F, Sun H, Wu L, Li B, Zhang B, Wang X, Yang L. TOP2A Promotes Lung Adenocarcinoma Cells’ Malignant Progression and Predicts Poor Prognosis in Lung Adenocarcinoma. J Cancer. 2020; 11:2496–508. 10.7150/jca.41415 32201520 PMC7066024

[31] Wang T, Lu J, Wang R, Cao W, Xu J. TOP2A promotes proliferation and metastasis of hepatocellular carcinoma regulated by miR-144-3p. J Cancer. 2022; 13:589–601. 10.7150/jca.64017 35069905 PMC8771514

[32] Ma S, Meng Z, Chen R, Guan KL. The Hippo Pathway: Biology and Pathophysiology. Annu Rev Biochem. 2019; 88:577–604. 10.1146/annurev-biochem-013118-111829 30566373

[33] Dey A, Varelas X, Guan KL. Targeting the Hippo pathway in cancer, fibrosis, wound healing and regenerative medicine. Nat Rev Drug Discov. 2020; 19:480–94. 10.1038/s41573-020-0070-z 32555376 PMC7880238

[34] Yang W, Han W, Qin A, Wang Z, Xu J, Qian Y. The emerging role of Hippo signaling pathway in regulating osteoclast formation. J Cell Physiol. 2018; 233:4606–17. 10.1002/jcp.26372 29219182

[35] Li C, Wang S, Xing Z, Lin A, Liang K, Song J, Hu Q, Yao J, Chen Z, Park PK, Hawke DH, Zhou J, Zhou Y, et al. A ROR1-HER3-lncRNA signalling axis modulates the Hippo-YAP pathway to regulate bone metastasis. Nat Cell Biol. 2017; 19:106–19. 10.1038/ncb3464 28114269 PMC5336186

[36] Wang J, Rouse C, Jasper JS, Pendergast AM. ABL kinases promote breast cancer osteolytic metastasis by modulating tumor-bone interactions through TAZ and STAT5 signaling. Sci Signal. 2016; 9:ra12. 10.1126/scisignal.aad3210 26838548 PMC4991033

[37] Oura K, Morishita A, Tani J, Masaki T. Tumor Immune Microenvironment and Immunosuppressive Therapy in Hepatocellular Carcinoma: A Review. Int J Mol Sci. 2021; 22:5801. 10.3390/ijms22115801 34071550 PMC8198390

[38] Ruf B, Heinrich B, Greten TF. Immunobiology and immunotherapy of HCC: spotlight on innate and innate-like immune cells. Cell Mol Immunol. 2021; 18:112–27. 10.1038/s41423-020-00572-w 33235387 PMC7852696

[39] Pinato DJ, Guerra N, Fessas P, Murphy R, Mineo T, Mauri FA, Mukherjee SK, Thursz M, Wong CN, Sharma R, Rimassa L. Immune-based therapies for hepatocellular carcinoma. Oncogene. 2020; 39:3620–37. 10.1038/s41388-020-1249-9 32157213 PMC7190571

[40] Xiang ZL, Zeng ZC, Tang ZY, Fan J, He J, Zeng HY, Zhu XD. Potential prognostic biomarkers for bone metastasis from hepatocellular carcinoma. Oncologist. 2011; 16:1028–39. 10.1634/theoncologist.2010-0358 21665914 PMC3228144

[41] Goldman MJ, Craft B, Hastie M, Repečka K, McDade F, Kamath A, Banerjee A, Luo Y, Rogers D, Brooks AN, Zhu J, Haussler D. Visualizing and interpreting cancer genomics data via the Xena platform. Nat Biotechnol. 2020; 38:675–8. 10.1038/s41587-020-0546-8 32444850 PMC7386072

[42] Wei L, Jin Z, Yang S, Xu Y, Zhu Y, Ji Y. TCGA-assembler 2: software pipeline for retrieval and processing of TCGA/CPTAC data. Bioinformatics. 2018; 34:1615–7. 10.1093/bioinformatics/btx812 29272348 PMC5925773

[43] GTEx Consortium. The Genotype-Tissue Expression (GTEx) project. Nat Genet. 2013; 45:580–5. 10.1038/ng.2653 23715323 PMC4010069

[44] Zhang J, Bajari R, Andric D, Gerthoffert F, Lepsa A, Nahal-Bose H, Stein LD, Ferretti V. The International Cancer Genome Consortium Data Portal. Nat Biotechnol. 2019; 37:367–9. 10.1038/s41587-019-0055-9 30877282

[45] Nusinow DP, Szpyt J, Ghandi M, Rose CM, McDonald ER 3rd, Kalocsay M, Jané-Valbuena J, Gelfand E, Schweppe DK, Jedrychowski M, Golji J, Porter DA, Rejtar T, et al. Quantitative Proteomics of the Cancer Cell Line Encyclopedia. Cell. 2020; 180:387–402.e16. 10.1016/j.cell.2019.12.023 31978347 PMC7339254

[46] Uhlen M, Oksvold P, Fagerberg L, Lundberg E, Jonasson K, Forsberg M, Zwahlen M, Kampf C, Wester K, Hober S, Wernerus H, Björling L, Ponten F. Towards a knowledge-based Human Protein Atlas. Nat Biotechnol. 2010; 28:1248–50. 10.1038/nbt1210-1248 21139605

[47] Li T, Fan J, Wang B, Traugh N, Chen Q, Liu JS, Li B, Liu XS. TIMER: A Web Server for Comprehensive Analysis of Tumor-Infiltrating Immune Cells. Cancer Res. 2017; 77:e108–10. 10.1158/0008-5472.CAN-17-0307 29092952 PMC6042652

[48] Ru B, Wong CN, Tong Y, Zhong JY, Zhong SSW, Wu WC, Chu KC, Wong CY, Lau CY, Chen I, Chan NW, Zhang J. TISIDB: an integrated repository portal for tumor-immune system interactions. Bioinformatics. 2019; 35:4200–2. 10.1093/bioinformatics/btz210 30903160

