# Supplementary material for: Identification of topoisomerase 2A as a novel bone metastasis-related gene in liver hepatocellular carcinoma
Source: Aging (Albany NY). 2023 Nov 17;15(22):13010–40. doi: 10.18632/aging.205216 (PMC10713393; doi:10.18632/aging.205216)
Supplement: Supplementary Figures [file aging-15-205216-s001.pdf]

SUPPLEMENTARY FIGURES

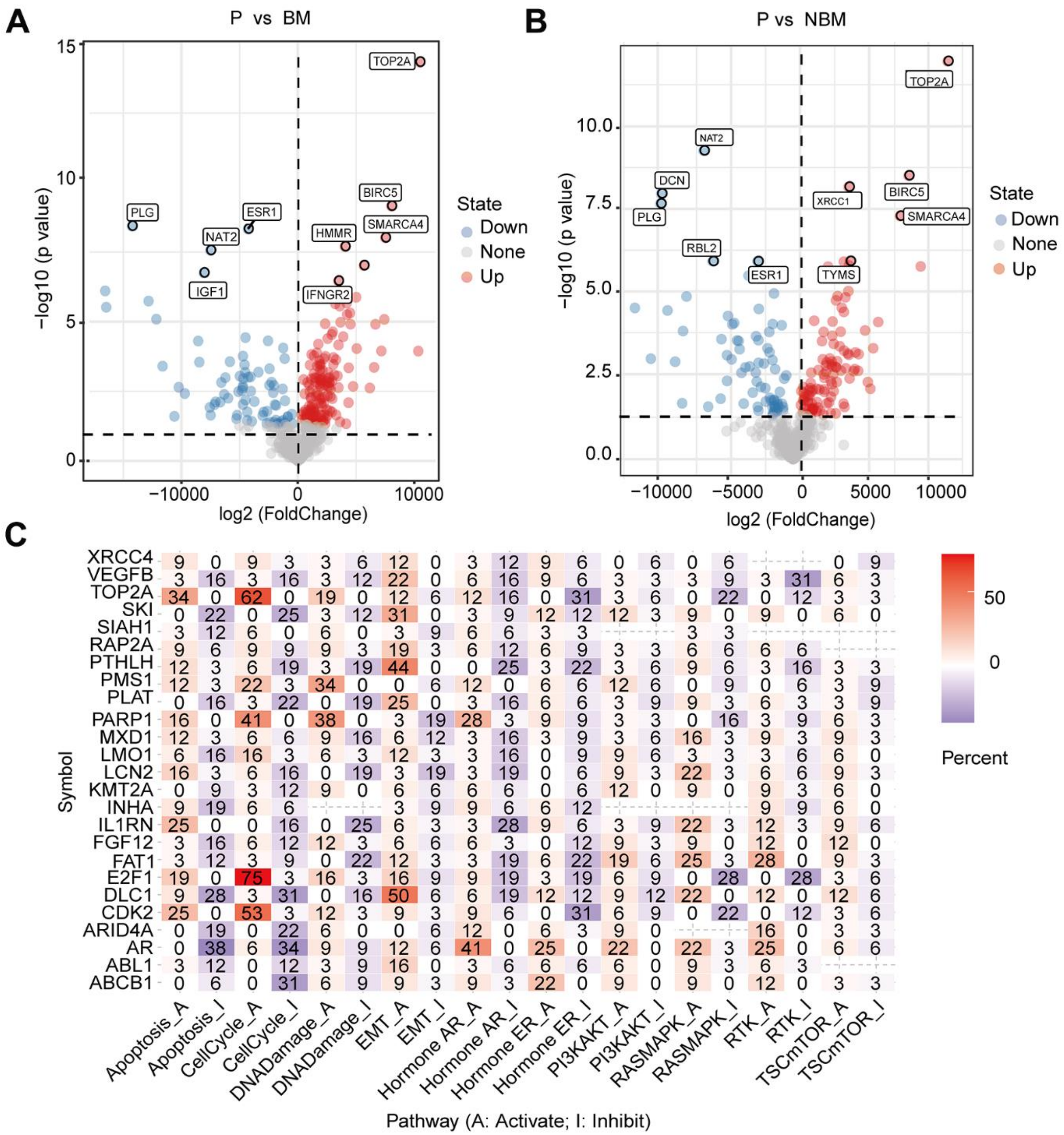

Supplementary Figure 1. DEGs between LIHC with bone metastasis (BM) and paracancerous tissues. (**A**) Volcano plot of DEGs between LIHC with BM and paracancerous tissues. (**B**) Volcano plot of DEGs between LIHC without BM and paracancerous tissues. (**C**) Pathway analysis of the 25 overlapping genes.

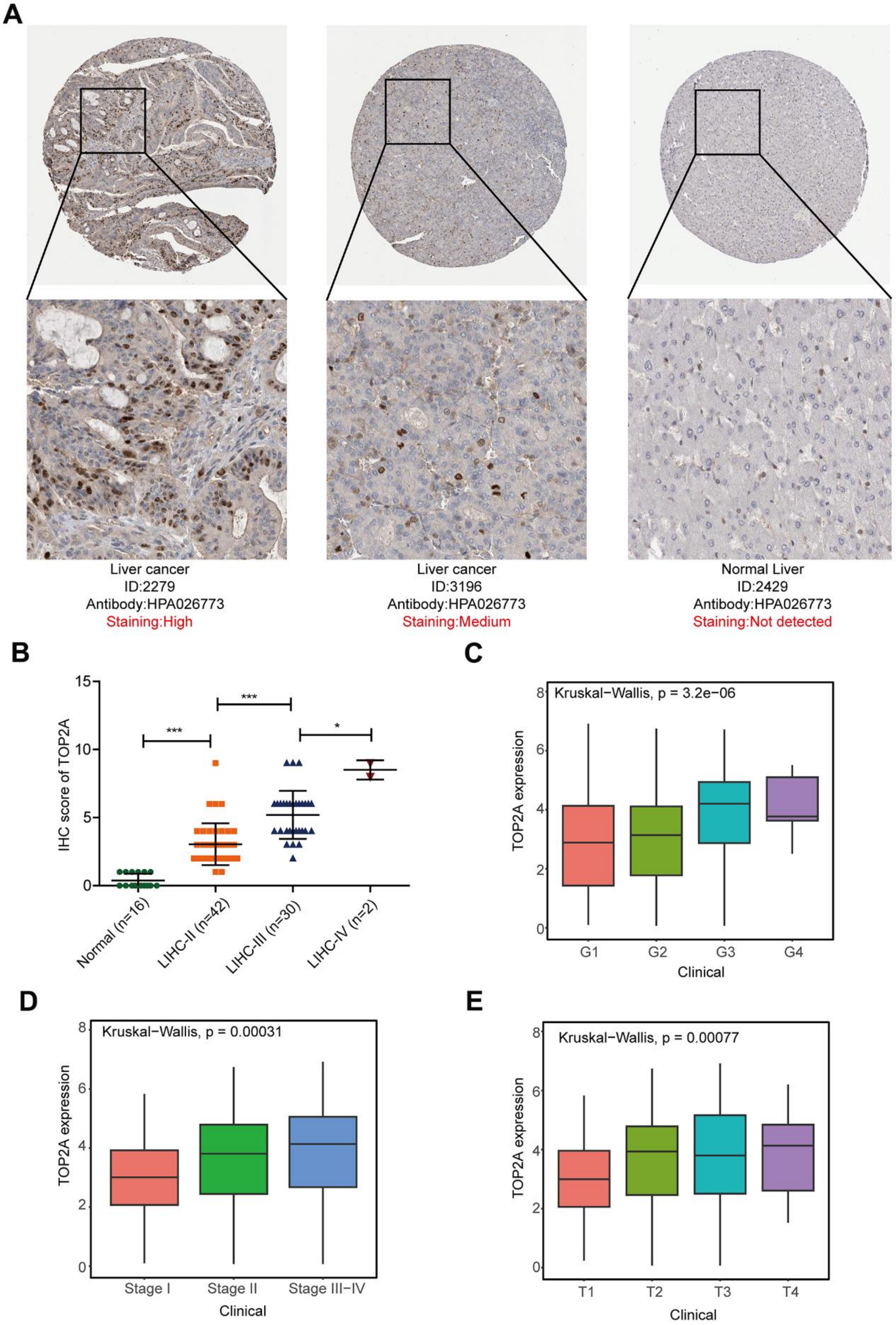

Supplementary Figure 2. The expression of TOP2A in LIHC. (**A**) The representative protein expression of TOP2A of LIHC tissue and normal liver tissue in HPA database. Magnification: 100 × (upper), and 400× (lower). (**B**) IHC score of TOP2A expression in LIHC with different stage and normal liver tissues from tissue microarrays. (**C**–**E**) TOP2A expression levels in patients from TCGA database were stratified into groups by (**C**) histological grade (**D**) clinical stage and (**E**) T stage.

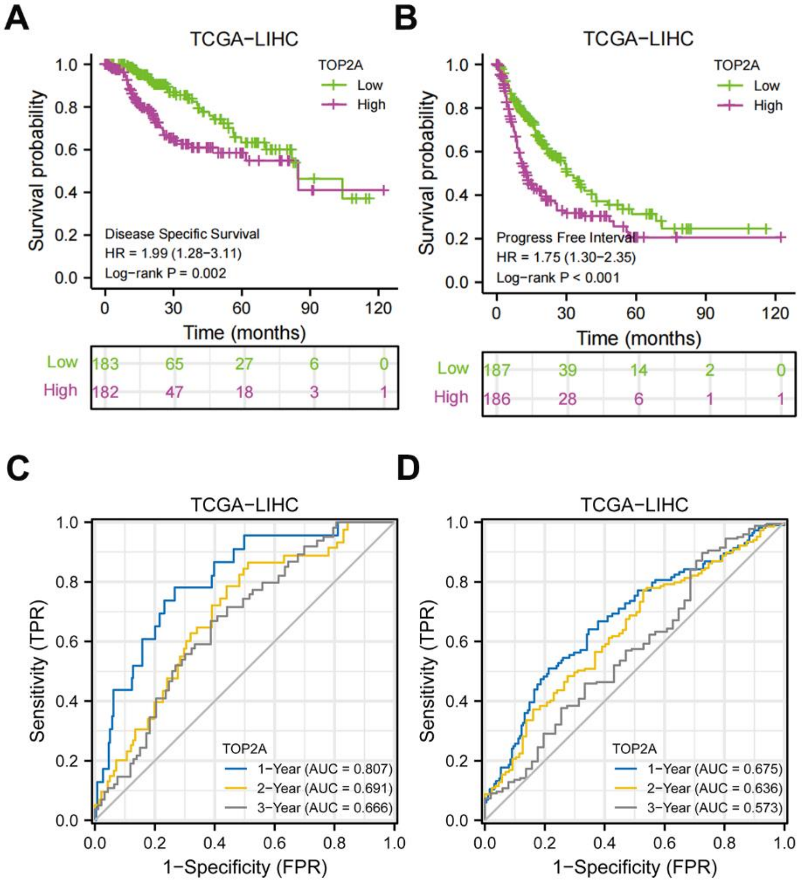

Supplementary Figure 3. The survival analysis of TOP2A in LIHC. (**A**) The disease-specific survival curve of TOP2A in LIHC. (**B**) The progress-free interval curve of TOP2A in LIHC. (**C**) ROC curves of TOP2A to predict the sensitivity and specificity of 1-, 2-, and 3-year disease-specific survival. (**D**) ROC curves of TOP2A to predict the sensitivity and specificity of the progress-free interval curve.

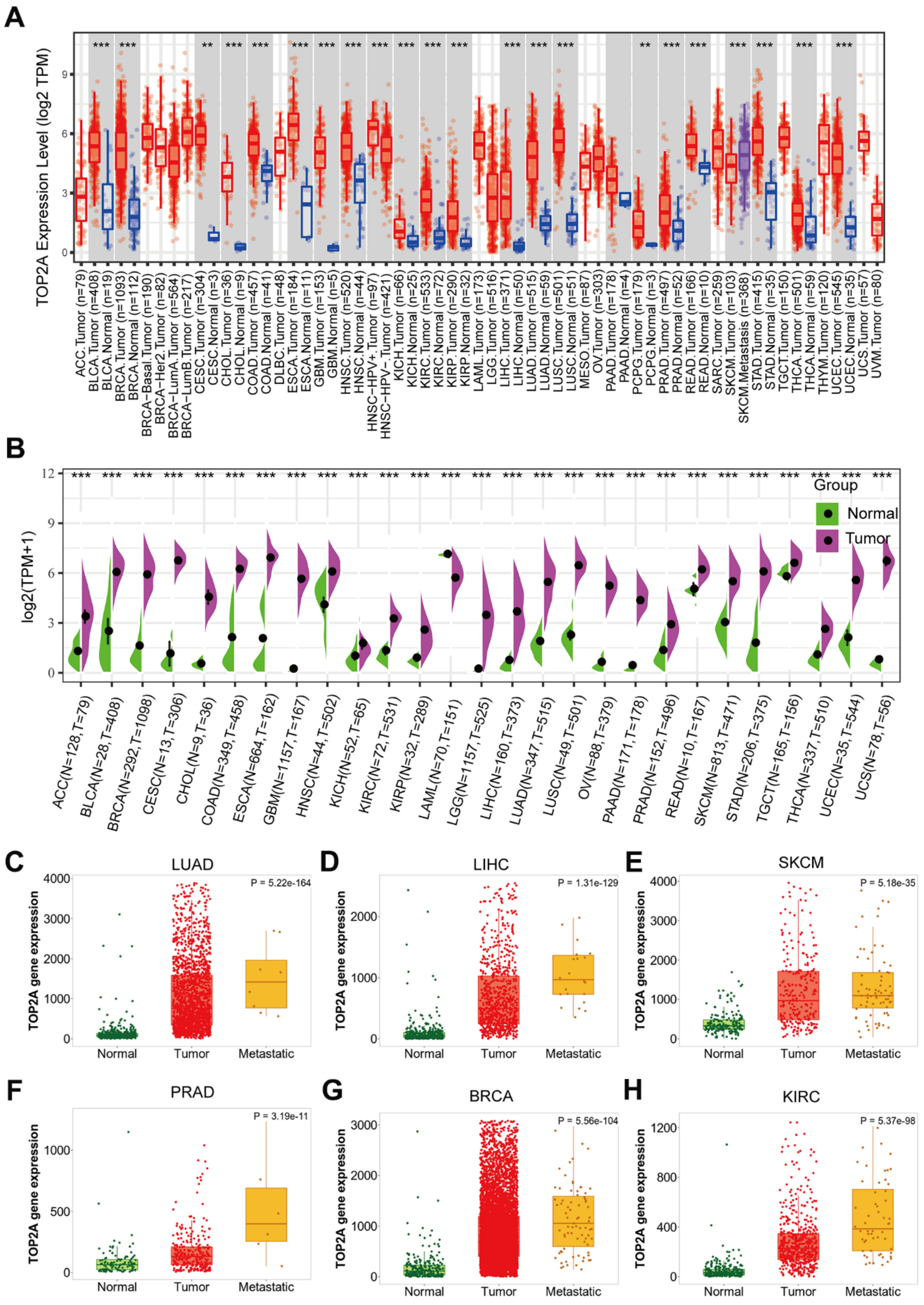

Supplementary Figure 4. TOP2A expression levels in human cancers. (**A**) The transcription levels of TOP2A in different cancer types and normal tissues from TCGA database. The color red represents for over-expression and blue for under-expression. (**B**) Expression levels of TOP2A in different types of cancers from TCGA and GTEx database. (**C**–**H**) Correlation analysis of TOP2A expression with metastasis in different types of cancers from GEO database. T, tumor; N, normal. $^{**}P < 0.01$, and $^{***}P < 0.001$.

CancerCode	pvalue	Hazard Ratio(95%CI)
TCGA-KIRP(N=276)	6.3e-14	2.10(1.71,2.59)
TCGA-LGG(N=474)	5.1e-13	1.50(1.34,1.68)
TCGA-KIRC(N=515)	1.2e-10	1.63(1.41,1.89)
TCGA-MESO(N=84)	7.3e-10	2.20(1.71,2.84)
TCGA-KICH(N=64)	8.1e-10	2.26(1.61,3.19)
TCGA-ACC(N=77)	1.8e-8	2.17(1.63,2.90)
TCGA-LIHC(N=341)	1.3e-5	1.31(1.16,1.48)
TCGA-PAAD(N=172)	1.7e-4	1.51(1.22,1.88)
TCGA-LUAD(N=490)	3.3e-3	1.17(1.05,1.30)
TCGA-UVM(N=74)	6.5e-3	2.02(1.20,3.37)
TCGA-SARC(N=254)	0.05	1.17(1.00,1.38)
TCGA-CHOL(N=33)	0.08	1.51(0.94,2.42)
TCGA-PRAD(N=492)	0.23	1.45(0.79,2.65)
TCGA-PCPG(N=170)	0.25	1.72(0.69,4.29)
TCGA-THCA(N=501)	0.30	1.37(0.75,2.50)
TCGA-SKCM(N=444)	0.33	1.07(0.93,1.22)
TCGA-ESCA(N=175)	0.49	1.07(0.89,1.27)
TCGA-GBM(N=144)	0.55	1.05(0.90,1.23)
TCGA-UCEC(N=166)	0.70	1.07(0.76,1.49)
TCGA-BRCA(N=1044)	0.86	1.01(0.90,1.13)
TCGA-HNSC(N=509)	0.89	1.01(0.87,1.17)
TCGA-THYM(N=117)	3.7e-3	0.62(0.44,0.88)
TCGA-CESC(N=273)	0.03	0.67(0.47,0.97)
TCGA-OV(N=407)	0.22	0.93(0.82,1.05)
TCGA-LAML(N=144)	0.27	0.89(0.71,1.10)
TCGA-STAD(N=372)	0.28	0.93(0.82,1.06)
TCGA-TGCT(N=128)	0.28	0.35(0.05,2.37)
TCGA-LUSC(N=468)	0.31	0.93(0.81,1.07)
TCGA-READ(N=90)	0.36	0.71(0.35,1.48)
TCGA-UCS(N=55)	0.42	0.85(0.56,1.27)
TCGA-BLCA(N=398)	0.53	0.96(0.83,1.10)
TCGA-COAD(N=278)	0.56	0.90(0.64,1.27)
TCGA-DLBC(N=44)	0.70	0.81(0.28,2.37)

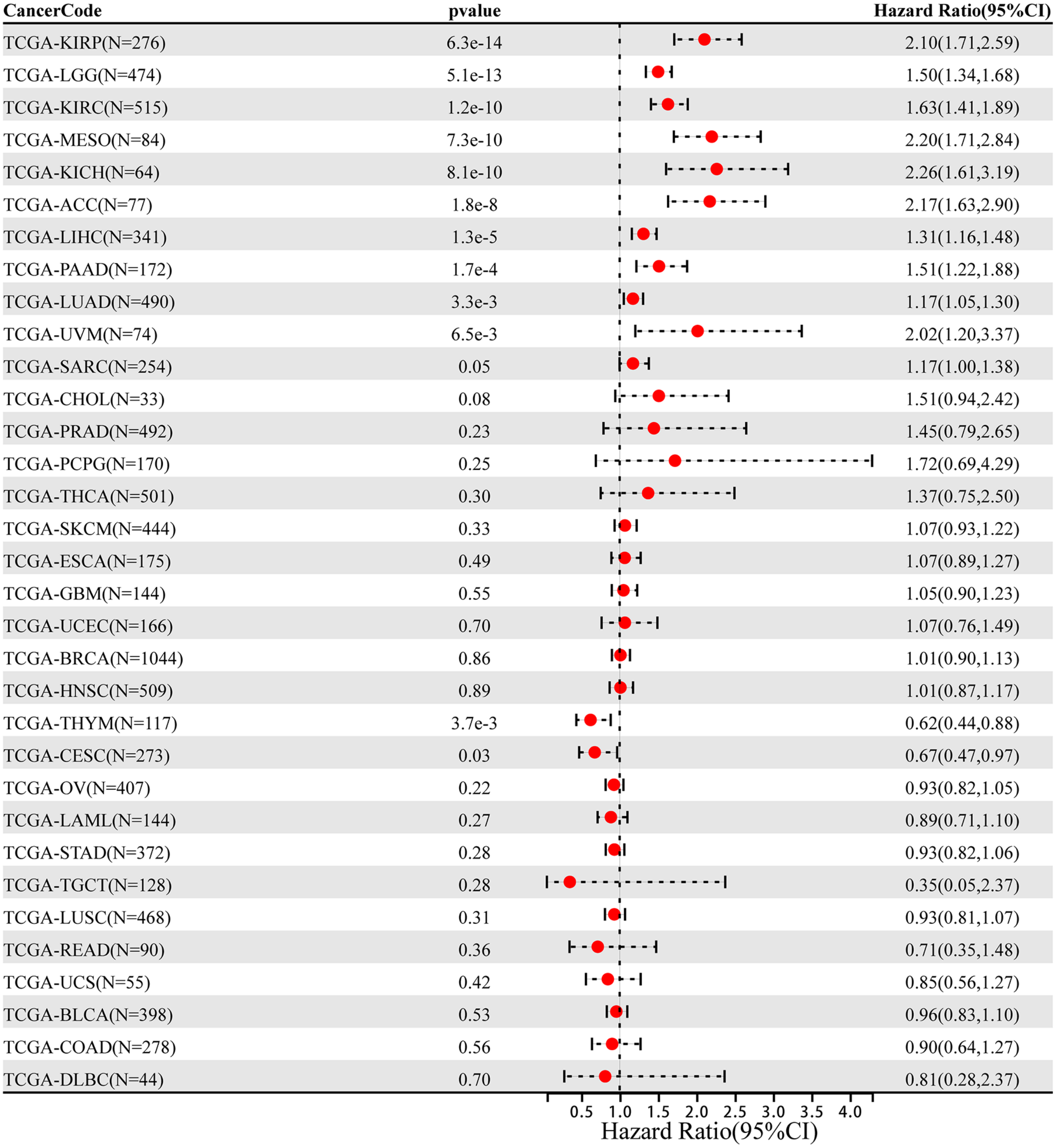

Supplementary Figure 5. The overall survival (OS) analysis of TOP2A in pan-cancer from TCGA.

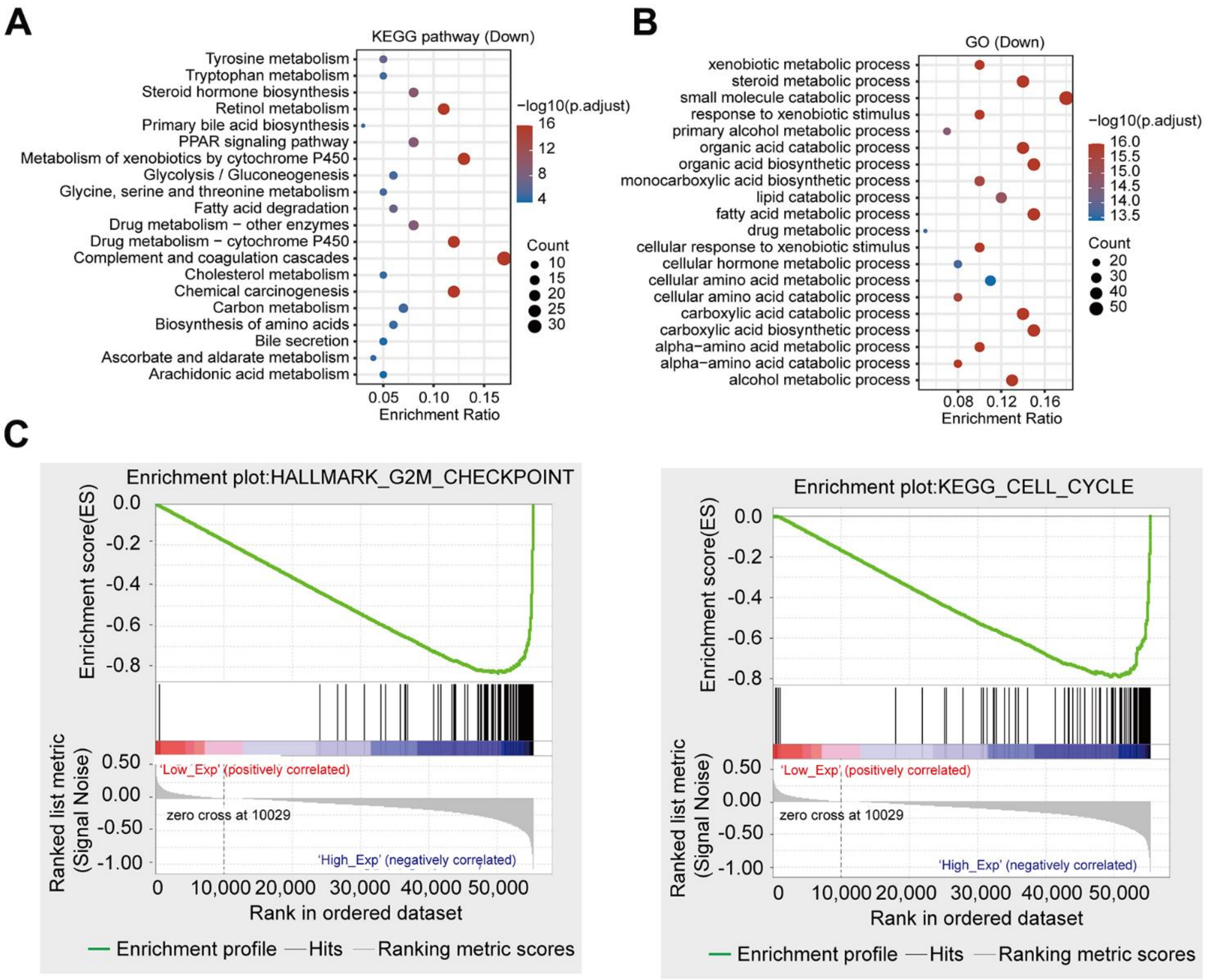

Supplementary Figure 6. Enrichment analysis of TOP2A in LIHC. (**A**, **B**) KEGG and GO enrichment analysis for the down-regulated genes in LIHC. (**C**) Cell cycle pathway in LIHC with high TOP2A expression by GSEA enrichment analysis.

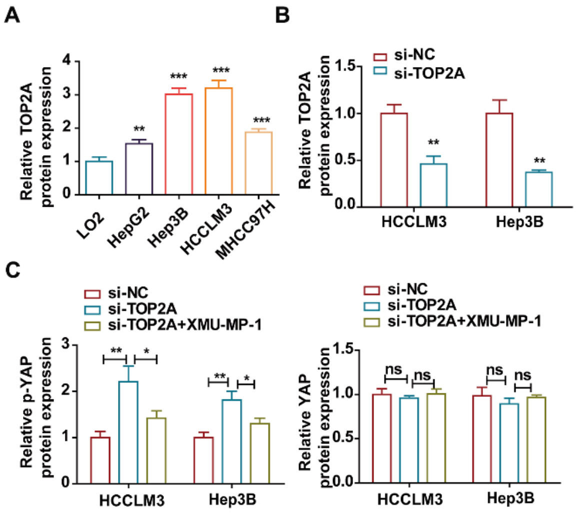

Supplementary Figure 7. TOP2A promotes tumor proliferation and metastasis via Hippo-YAP pathway in HCCLM3 and Hep3B cells. (**A**) The relative protein expression of TOP2A in normal liver cell line and four liver cancer cell lines. (**B**) The knockdown efficiency of TOP2A siRNA was determined by Western blot in HCCLM3 and Hep3B cells. (**C**) The relative protein expression of p-YAP and YAP was detected by Western blot in the indicated groups of HCCLM3 and Hep3B cells. β-actin was used as a control. **P* < 0.05, ****P* < 0.001, *****P* < 0.0001. ns, not significant. Data presented as mean ± SD. Experiments were repeated at least three times.
